# Targeting small GTPases: emerging grasps on previously untamable targets, pioneered by KRAS

**DOI:** 10.1038/s41392-023-01441-4

**Published:** 2023-05-23

**Authors:** Guowei Yin, Jing Huang, Johnny Petela, Hongmei Jiang, Yuetong Zhang, Siqi Gong, Jiaxin Wu, Bei Liu, Jianyou Shi, Yijun Gao

**Affiliations:** 1 The Seventh Affiliated Hospital of Sun Yat-sen University, Shenzhen, 518107 China; 2 State Key Laboratory of Oncology in South China, Guangdong Key Laboratory of Nasopharyngeal Carcinoma Diagnosis and Therapy, Sun Yat-sen University Cancer Center, Guangzhou, 510060 China; 3 Wake Forest University School of Medicine, Winston-Salem, NC 27101 USA; 4 School of Medicine, Sun Yat-Sen University, Shenzhen, 518107 China; 5 National Biomedical Imaging Center, School of Future Technology, Peking University, Beijing, 100871 China; 6 Department of Pharmacy, Personalized Drug Therapy Key Laboratory of Sichuan Province, Sichuan Academy of Medical Sciences & Sichuan Provincial People’s Hospital, School of Medicine, University of Electronic Science and Technology, Chengdu, 610072 China

**Keywords:** Molecular medicine, Drug discovery

## Abstract

Small GTPases including Ras, Rho, Rab, Arf, and Ran are omnipresent molecular switches in regulating key cellular functions. Their dysregulation is a therapeutic target for tumors, neurodegeneration, cardiomyopathies, and infection. However, small GTPases have been historically recognized as “undruggable”. Targeting KRAS, one of the most frequently mutated oncogenes, has only come into reality in the last decade due to the development of breakthrough strategies such as fragment-based screening, covalent ligands, macromolecule inhibitors, and PROTACs. Two KRAS^G12C^ covalent inhibitors have obtained accelerated approval for treating KRAS^G12C^ mutant lung cancer, and allele-specific hotspot mutations on G12D/S/R have been demonstrated as viable targets. New methods of targeting KRAS are quickly evolving, including transcription, immunogenic neoepitopes, and combinatory targeting with immunotherapy. Nevertheless, the vast majority of small GTPases and hotspot mutations remain elusive, and clinical resistance to G12C inhibitors poses new challenges. In this article, we summarize diversified biological functions, shared structural properties, and complex regulatory mechanisms of small GTPases and their relationships with human diseases. Furthermore, we review the status of drug discovery for targeting small GTPases and the most recent strategic progress focused on targeting KRAS. The discovery of new regulatory mechanisms and development of targeting approaches will together promote drug discovery for small GTPases.

## Introduction

**Fig. 1:**
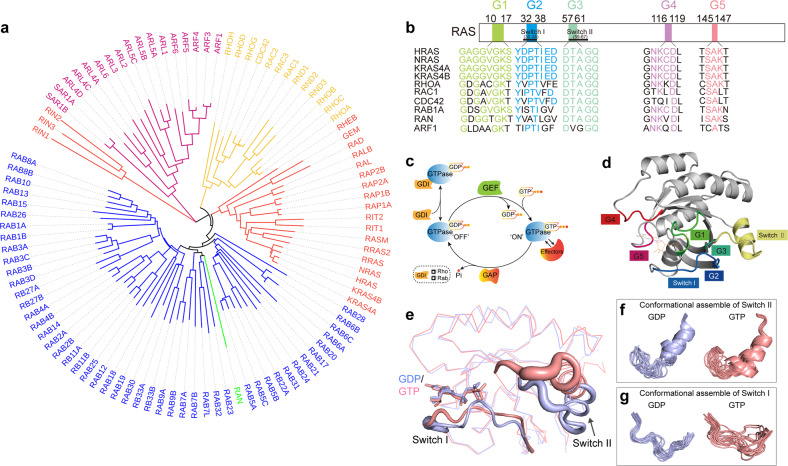
Overview of small GTPases biology. Phylogenetic tree (**a**), and five conserved boxes (G1–G5) (**b,** **d**), of Ras, Rho, Rab, Arf and Ran families. **c** Molecular switch diagram: GEF-mediated GDP-GTP exchange for activation, GAP-mediated GTP hydrolysis for deactivation, GDI stabilizes the GDP-bound form of Rho and Rab proteins, and the GTP-bound small GTPases interact with effector proteins. **e** Structural difference of Switch regions between GDP-state (PDB: 4LPK, light purple) and GTP-bound state (PDB: 6GOD, pink). Conformational ensembles of Switch II (**f**), and Switch I (**g**), regions of HRAS are generated from NMR structures (PDB: 1CRP and 2LCF)

Small GTPases are a large family of low molecular weight enzymes that hydrolyze GTP. It contains more than 150 members, which can be divided into five families: Rat Sarcoma (Ras), Rhodopsin (Rho), Ras-related in brain (Rab), ADP-ribosylation factor (Arf), and Ras-related nuclear protein (Ran), on the basis of their sequence homology and physiological functions [Fig. 1a]. Small GTPases play essential roles in regulating a wide range of cellular activities including cell survival, cell cycle progression, proliferation, apoptosis, differentiation, adhesion and migration as well as subcellular events such as cytoskeletal dynamics and vesicle trafficking. They serve as key molecular switches that mediate cellular processes through recycling between GTP-bound state “active (*on*)” and GDP-bound state “inactive (*off*)” [Fig. 1c]. Generally, guanine nucleotide exchange factors (GEFs) bind to the GDP-bound small GTPases and facilitate the exchange from GDP to GTP,^1^ which induces conformational change that permits binding of effectors to produce distinct signal outputs. Conversely, GTPase-activating proteins (GAPs) terminate small GTPases signaling by stimulating GTP hydrolysis.^2–4^ For Rho and Rab family members, there is another class of proteins which regulate the activation of small GTPases: guanine nucleotide dissociation inhibitors (GDI).^5^ They bind to GDP-bound forms of GTPases, preventing GDP dissociation and maintaining their inactive state.^6^ The release of GDIs from these small GTPases prior to GDP-GTP exchange is tightly regulated by GDI displacement factors.^7^

Ras family is the root of the small GTPase superfamily according to the phylogenetic reconstruction analysis, so the small GTPase family is also known as Ras superfamily.^8^ Ras family consists of 36 members falling into seven subfamilies: Ras (KRAS4A, KRAS4B, NRAS, and HRAS), Ral, Rheb, Rap, Rad, Rit and DIRAS, which serve as central nodes of a wide range of signaling pathways controlling cell survival, proliferation, differentiation, migration, and adhesion.^9,10^ As the first human oncogene to be identified, Ras has been well established as an oncogenic driver in multiple human cancers. Hotspot mutations in Ras drastically impair its GAP-mediated GTP hydrolysis activity, lead to constitutive activation of Ras and drive cell transformation and tumor initiation in different cancer models. Germline mutations of Ras result in aberrant activation of the downstream Mitogen-Activated Protein Kinase (MAPK) pathway and cause developmental disorders in patients, known as RASopathies. In addition, new members of Ras subfamily are being discovered through sequence homology screen and their functions are distinct from well-characterized Ras subfamily members like HRAS, KRAS, and NRAS.^11^ For example, DIRAS subgroup, consisting of DIRAS-1, DIRAS-2, and DIRAS-3 (Noey2), were found to be downregulated in cancer and act as tumor suppressors.^12–15^ Their biochemical properties and physiological functions remain to be further elucidated prior to being established as therapeutic targets of tumors. The Rho family comprises 22 members including Rho-related proteins (RhoA) subfamily, cell division control protein 42 homolog (Cdc42) subfamily, and Ras-related C3 botulinum toxin substrate (Rac) subfamily that are involved in cytoskeletal organization.^16^ Dysregulation of the key members within Rho family, such as RhoA and Cdc42, facilitates tumor progression via promoting epithelial to mesenchymal transition (EMT) and invasion of tumor cells.^17,18^ Mutations of Rho family members also have been linked to the development of neurologic and vascular diseases.^19–21^ Rab, the largest family of the small GTPases with more than 60 members, regulates membrane trafficking between different intracellular organelles and the plasma membrane.^22,23^ Aberrant regulation of Rab-mediated vesicle trafficking promotes tumor progression through enhancing cell migration/invasion.^24–29^ Rab mutations have also been implicated in neurologic diseases due to dysfunctional membrane trafficking.^30,31^ Arf is the fourth family of this superfamily, containing six members that are involved in several processes of membrane trafficking and tumor cell migration.^32^ Additionally, lack of ARF may cause brain diseases due to insufficient neuron migration.^33^ Finally, Ran family, the most abundant protein in cells with only one recognized member, is involved in nuclear transport.^34^ Ran GEFs interact with Ran in the nucleus, allowing GTP–bound Ran to bind to and transfer its client proteins from nucleus to cytoplasm.^35^ GTP-bound Ran is subsequently dissociated and inactivated by the engagement of Ran GAPs located in the cytoplasm. Ran-mediated nuclear-cytoplasmic transport accelerates the cell cycle and efficiency of DNA repair, facilitating proliferation in tumor cells. Additionally, defective Ran has been found in the development of Alzheimer’s disease and other brain diseases.^36^

Despite being famous therapeutic targets in human diseases, small GTPases have been termed as “undruggable” for decades, mainly owing to the absence of pharmacologically targetable pockets within wild types and mutant isoforms. Early efforts were devoted to developing indirect targeting strategies that interfere with GEFs mediated activation of small GTPases. As the most frequently mutated oncogene in human cancer, KRAS has been established as a paradigm for targeting Ras proteins and other small GTPases. Extensive studies have been focused on targeting KRAS post-translational modifications, membrane trafficking, upstream regulators (e.g., GEFs, GAPs), downstream effectors (e.g., MEK-ERK signaling, PI3K-AKT signaling), synthetic lethal partners and metabolic alterations. However, single agent treatment targeting the above targets have had insufficient efficiency in *RAS*-mutant cancer patients, and combinatorial targeting its downstream pathways also failed due to severe clinical toxicity. With emerging new targeting approaches and improvements in covalent drug design, the success of Sotorasib, an allele-specific inhibitor of KRAS^G12C^, finally marked the dawn of a new era in targeting small GTPases directly. Inspired by the accelerated approval of Sotorasib for the treatment of *KRAS*^*G12C*^ mutant NSCLC, an upsurge in the development of KRAS inhibitors has spawned many new cutting-edge strategies, such as fragment-based screening, macromolecular inhibitors, PROTACs, genetic targeting and antibodies targeting covalent inhibitor based neoepitopes. Many more hotspot mutant alleles of KRAS including G12D/S/R have proven to be targetable in the past few years, providing valuable insights into the development of inhibitors of remaining KRAS mutant alleles and other Ras isoforms.^37–39^ Despite the rapid progress made in targeting KRAS during the past decade, hotspot mutations such as G12V, G13, and Q61 in KRAS, as well as Q61R/L/H in NRAS, still lack targeting methods. With the successful clinical application of KRAS^G12C^ inhibitors in NSCLC, the rapid onset of drug resistance driven by heterogenous mechanisms remains a major inevitable and urgent challenge that needs to be addressed to maximize clinical benefits. Moreover, development of selective inhibitors targeting other members of small GTPases is lagging, and the vast majority of small GTPases remain untargeted.

In this review, we provide an overview for structural properties, physiological functions, post-translational modifications, and regulation of small GTPases that underpin their fundamental roles as molecular switches in cells. Pathologic dysregulations of small GTPases in various human diseases including tumors, neurodegenerative diseases, cardiomyopathies, and infections are summarized. More importantly, we review the recent strategic developments in targeting small GTPases directly and indirectly, highlighting the breakthrough targeting strategies of KRAS and discussing crosstalk between Ras and Rho/Ran family proteins in tumorigenesis. We hope that this review will lay the groundwork for developing GTPases-based cooperative targeting. Next, we summarize mechanisms responsible for resistance of KRAS^G12C^ inhibitors in clinical trials and discuss potential combination strategies to prevent or delay resistance. In the last session, we discuss the future directions for targeting small GTPases based on the most recent outcomes in clinical trials and drug resistance studies, new regulatory mechanisms, and new targeting strategies.

## Small GTPases biology

### Structural basis for molecular switch

The primary structure of small GTPases is divided into two segments, G-domain (GTP-binding domain) for nucleotide-binding and C-terminal hypervariable region for membrane association. In 1988, the crystal structures of HRAS G-domain in complex with GDP/GTP were determined, which served as a prototype to understand structural properties shared among different small GTPases in Ras superfamily.^40^ Despite sequence divergences, small GTPases adopt canonical Rossmann fold composed of six β-sheets and five α-helices. There are five conserved motifs termed as G1–G5 representing five important loops for nucleotide binding and structural regulations^41,42^ [Fig. 1b, d]. G1 (residue 10–16 in Ras) is the first loop in the protein and participates in binding to nucleotide phosphates. Therefore, G1 is referred to as the P-loop, and it contains two oncogenic mutation hotspots, G12 and G13, in KRAS, as well as the G12V mutation in Cdc42.^43,44^ G2 (residue 32–38 in Ras) consists of the majority of Switch I, Thr35 (Thr35 in Ras, Rac1, and Cdc42 and Thr37 in RhoA) located in Switch I is a conserved residue for interacting with γ-phosphate and magnesium ion.^45^ As such, Switch I exhibits structural plasticity directly related to the nucleotide binding. G3 (residue 57–61 in Ras) spans across part of Switch II and helix 2. G3 harbors another oncogenic hotspot, Q61, which is frequently mutated in NRAS, HRAS, and Cdc42 because the sidechain of Q61 plays an important role in GTP-hydrolysis.^43,44^ G4 (residue 116–119 in Ras) and G5 (residue 145–147) are two loop regions for anchoring guanine bases and ribose and dictating the binding selectivity to guanosine over adenosine (ATP/ADP).^46^ In G4, K117 interacts with ribose, and its disease-related mutations (K117N and K117R) are characterized as “fast-cycling” because the weakened binding between protein and ribose of guanine nucleotide leads to higher exchange rates between bound and unbound nucleotides.^47,48^ Both K117 and K147 serve as hotspots for PTMs in the G-domain of Ras. Beyond the intramolecular interactions that involve the G1-G5 motifs, Switch I (residue 30–38) [Fig. 1e, g] and Switch II (residue 59–67) [Fig. 1e, f] are two structural regions that underpin the function of molecular switches for small GTPases. They possess significant structural plasticity by changing their conformations between GDP- and GTP-bound states.^42^ The different conformations of Switch regions dictate the specificity of small GTPases to interact with different regulatory proteins (GEFs, GAPs, GDIs) and downstream effectors. Moreover, the switch regions are highly dynamic and present as structural ensembles containing multiple coexisting and interconverting conformations. Both structure and dynamics of switch II are regulated by nucleotide binding, which have been exploited as a structural basis for developing inhibitors.^49,50^

### Biochemistry of small GTPases: regulations and effector interactions

As the molecular switch, binary cycling between GDP’*OFF*’- and GTP’*ON*’-bound states is a signature process of small GTPases in regulating signal transduction [Fig. 1c]. The cycling is highly regulated by two major categories of regulatory proteins: GEFs and GAPs.^3^ To activate small GTPases, GEFs recognize the GDP-bound conformation of switch regions and facilitate the GDP-GTP exchange. In the GTP-bound state, small GTPases adopt distinct conformations in both Switch I and Switch II regions compared to the GDP-bound state [Fig. 1e–g]. Activated small GTPases interact with a variety of catalytically distinct downstream effectors such as RAF kinases and PI3K in Ras pathways. GAPs bind to the active small GTPases and catalyze the GTP hydrolysis through an arginine finger. Following this, the protein returns to the GDP-bound “OFF” state. Many oncogenic mutations impair GEF- and GAP-regulations; the GAP-deficiency due to disrupted GAP interactions with the mutant GTPases is characterized as a major consequence of the mutation to drive tumorigenesis. GEFs and GAPs can be shared within a small GTPases subfamily.^8^ Among different subfamilies, GEFs and GAPs are structurally distinct but mechanistically similar. In a recent study, a non-arginine finger GAP, RGS3, has been found to facilitate GTP hydrolysis of oncogenic KRAS^G12C^ mutant in tumor cell lines.^51^ Additionally, GDP-binding affinity is relatively weak for Rho and Rab families, and GDIs are required for maintaining their GDP-binding *inactive* state.^5^

### Signaling pathways

#### Ras

**Fig. 2:**
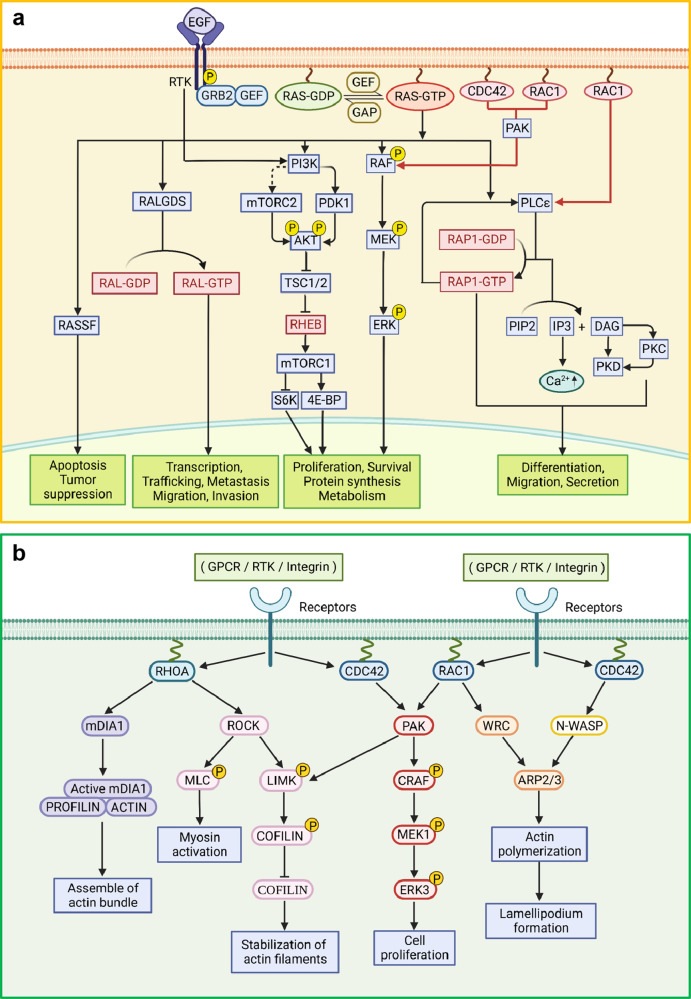
Cellular signaling coordinated by Ras and Rho subfamilies. **a** Ras-mediated signaling pathways include RAS-RAF-MEK-ERK, RAS-PI3K-AKT, RAS-RALGDS-RAL, RAS-PLCε, RAS-RASSF, the crosstalk between Ras, and other small GTPases including CDC42, RAC1 and RAP1, RHEB, and RAL are highlighted by red color. **b** Cytoskeletal dynamics are regulated by Rho, Rac and CDC42 through their main downstream signaling nodes such as ROCK, mDIA1, LIMK, PAK, WRC, and N-WASP. This figure was created with BioRender.com

Ras family proteins are coordinating or participating in multiple important signaling pathways in cells [Fig. 2a]. The intracellular signal transductions coordinated by RAS branch or RAS subfamily members (HRAS, NRAS, and KRAS) such as MAPK and PI3K-AKT, which are some of the most characterized pathways in research of cell biology and human diseases, especially in tumors. The MAPK pathway relaying cellular signal through RAS-RAF-MEK-ERK plays a critical role in cell cycle and proliferation. The MAPK cascade is initiated by activation of receptor tyrosine kinases (RTKs) upon extracellular ligand binding. The activated RTKs recruit growth factor receptor-bound protein 2 (GRB2) and RAS-GEF (SOS1). RAS-GEF then activates RAS by catalyzing GDP-GTP exchange, which switches RAS from its “*OFF*” to “*ON*” state. The activated RAS binds to RAF kinases and the signal is transduced downstream through RAF-MEK-ERK phosphorylation cascades and regulates a wide variety of cellular processes including cell proliferation, survival, cell cycle progression and differentiation. Ras proteins also regulate the PI3 Kinase (PI3K)-AKT-mTOR pathway, which plays an important role in regulating cell survival, proliferation, and motility.^52,53^ Both RAS and RTK can activate PI3K and recruit it to the plasma membrane; PI3K phosphorylates lipid molecule PIP2 to PIP3, and PIP3 triggers AKT phosphorylation by PDK1^54^ and mTORC2.^55,56^ The signal amplitude of PI3K-AKT is modulated by lipid phosphatase PTEN, which converts PIP3 to PIP2. As such, PTEN serves as a suppressor to tumorigenesis.^57,58^ RalGEF (RalGDS) is another downstream effector of RAS proteins and catalyzes the nucleotide exchange and activates another RAS family member, RAL. RAL GTPases located at a downstream signaling node in the RAS pathway engage with several downstream factors involved in transcription, trafficking, tumor cell invasion and metastasis.^59,60^ Phospholipase C (PLCε) is positioned at the center of another RAS-mediated pathway in regulating cell differentiation, migration, and secretion.^61,62^ PLCε contains a catalytic domain, two RAS-associating (RA) domains, and a GEF domain.^63^ In one mechanism, PLCε is activated by RAS and then hydrolyzes PIP2 to inositol 1,4,5-trisphosphate (IP3) and diacylglycerol (DAG). IP3 then induces the release of Ca^2+^ from the endoplasmic reticulum while DAG activates protein kinase C (PKC) and protein kinase D.^61,64,65^ In a different mechanism, PLCε mediates nucleotide exchange of another RAS family member, RAP1, and the activated RAP1 binds to one RA domain of PLCε and activates PLCε activity as a positive feedback loop.^63,66^ PLCε can also be activated by G protein-coupled receptor (GPCR)-G protein-RHO pathway.^67,68^ RAS-association domain family (RASSF) proteins are another group of RAS effectors; there are ten members identified and they contain Ras-association (RA) domain. RASSFs connect Ras to Hippo pathway in apoptosis (RASSF1-6) and p53 regulation in tumor suppression (RASSF7-10). Among these, RASSF1 and RASSF5 (NORE1A) are the best characterized members in terms of their interactions with Hippo kinase ortholog MST1 (mammalian sterile twenty).^69,70^

#### Rho

RHO GTPases play critical roles in modulating cytoskeletal dynamics [Fig. 2b]. RHO, RAC, and CDC42 are the three best characterized subfamilies. They modulate cell polarity, morphology, migration, and cell cycle progression.^71,72^ RND and RhoBTB are two other subfamilies. RND proteins include RNDs and RhoE and antagonize the RHO signaling in different tissues. However, the RhoBTB subfamily is still poorly characterized. RHO-associated coiled-coil containing kinase (ROCK), one of the downstream effectors of RHOA, phosphorylates MLC (myosin light chain) and leads to the activation of myosin, which is involved in cytoskeletal reorganization.^73–75^ ROCK also regulates actin assembly through ROCK-LIMK (LIM kinase)-COFILIN phosphorylation cascades. Interestingly, LIMK is also regulated by RAC1 and CDC42: the activated forms of both bind to PAK (p21-activated kinase) and enhance PAK/LIMK association, leading to increased phosphorylation of LIMK by PAK.^16,76^ RHOA-mDIA1-ACTIN is another RHOA pathway in which mDIA1, a formin family protein, (Diaphanous-related formin) mediates actin nucleation and elongation as well as stabilization of microtubules.

Ras-related C3 substrate (RAC) subfamily proteins mediate cell migration by affecting cytoskeletal dynamics. WASP family verprolin homologous protein (WAVE) and PAK are two well-characterized effectors of RAC1. RAC1 binds to WRC (WAVE regulatory complex) and subsequently actives ARP2/3 complex,^77^ which plays an important role in actin polymerization and formation of lamellipodia.^78,79^ PAK is a shared signaling node located downstream of both RAC1/CDC42 and RHOA. PAK-LIMK-COFILIN pathway is essential in stabilizing the structure of actin filaments, leading to the accumulation of these filaments at the leading edge of moving cells.^80^ Interestingly, CRAF, MEK1 and ERK3 are substrates of PAK, indicating PAK serves as a regulator of RAS-driven MAPK pathway.^81^ CDC42 regulates a wide spectrum of cellular processes including cell migration, polarity, transformation, division, and invasion. Besides PAK, Wiskott Aldrich syndrome protein (WASP) is another main CDC42 effector and the CDC42-bound N-WASP activates ARP2/3 complex in a similar manner as RAC1-bound WRC, meaning this pathway also regulates actin.^82,83^

There are several instances of crosstalk between RHO and RAS proteins.^84,85^ Activation of CDC42, RAC1, and RHOA is necessary for RAS-induced malignant transformation of fibroblasts.^86–89^ Loss of CDC42 in HRAS^G12V^-transformed cells leads to significant changes in morphology and inhibition of the cell cycle and proliferation.^85^ Constitutive activation of CDC42 prevents EGFR degradation and leads to sustained MAPK signaling.^90^ CDC42 activates PAK4 at the Golgi apparatus, which is important in RAS-mediated transformation.^91^

#### Rab

**Fig. 3:**
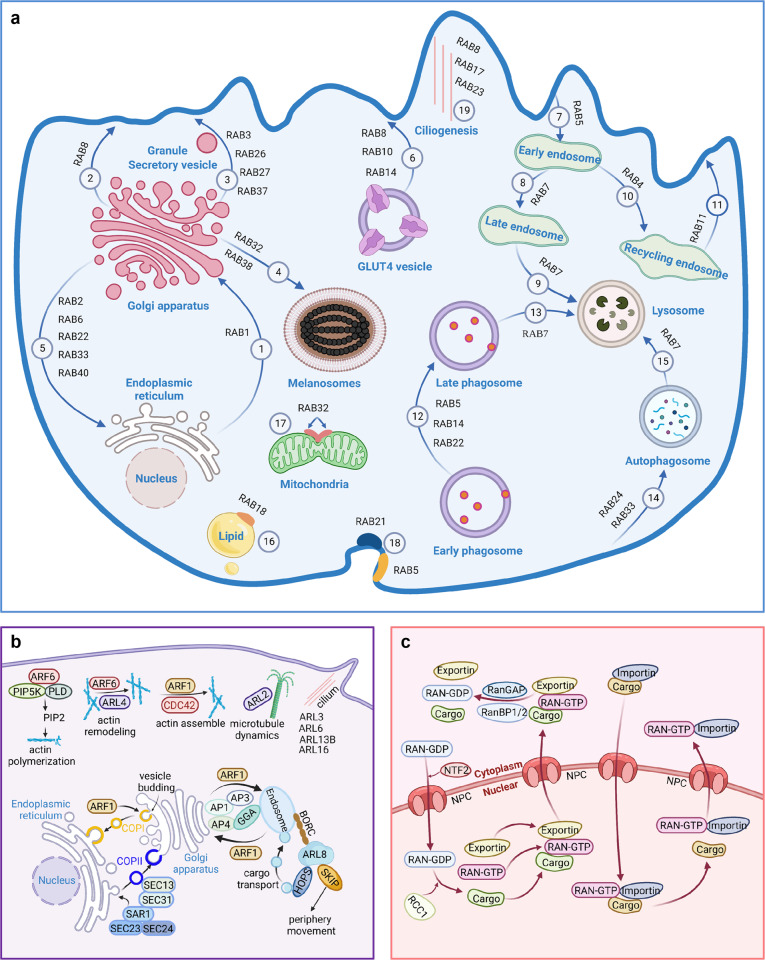
Cellular trafficking coordinated by Rab, Arf and Ran subfamilies. **a** Multiple vesicle trafficking steps are coordinated by Rab proteins: (1) ER to Golgi by RAB1, (2) Golgi to plasma membrane by RAB8, (3) Secretory vesicles and granules trafficking between Golgi and plasma membrane by RAB3, RAB26, RAB27 and RAB37, (4) Golgi to melanosome by RAB32 and RAB38 for biogenesis of melanosome, (5) Golgi to ER by RAB2, RAB6, RAB22, RAB33 and RAB40, (6) GLUT4 vesicle trafficking to plasma membrane by RAB8, RAB10 and RAB14, (7-9) Plasma membrane to early endosome (RAB5), then to late endosome (RAB7) and finally to lysosome (RAB7), (10-11) Early endosome to recycling endosome (RAB4), then to plasma membrane (RAB11), (12) Early phagosome to late phagosome by by RAB5, RAB14 and RAB22, (13) Late phagosome to lysosome by RAB7, (14-15) Autophagosome budding from plasma membrane by RAB24 and RAB33 and trafficking to lysosome by RAB7, (16) Lipid droplet biogenesis and homeostasis mediated by RAB18, (17) Mitochondrial fission mediated by RAB32, (18) Endocytosis by RAB5 and RAB21, (19) Ciliogenesis by RAB8, RAB17 and RAB23. **b** Vesicle trafficking and cytoskeletal dynamics are regulated by Arf proteins including ARF, ARL and SAR. **c**, Nucleocytoplasmic cargo transport through NPC is controlled by RAN. This figure was created with BioRender.com

Ras-like proteins in brain (RAB), ADP-ribosylation factor (ARF), and Ras-like nuclear (RAN) proteins are three families of small GTPases that are important in regulating vesicle trafficking (RAB and ARF) and nucleocytoplasmic transport (RAN). The RAB proteins comprise the largest family of RAS superfamily with more than 60 members and each has its own effector proteins. Rab effectors include a wide range of proteins such as tether proteins (p115, GM130, Giantin, Golgin-84, Rabenosyn-5 etc.), regulatory proteins (Rabphilin-3, Rabip4 etc.), adapter proteins (Rabaptins, Rabankyrin-5, Tip47, etc.), motors/motor adapters, scaffold proteins, SNARE proteins, kinases and phosphatases. And the list of Rab effectors is growing with new Rab downstream proteins being identified.^23,92,93^ Through interactions with specific effectors, RABs coordinate multiple steps of membrane trafficking between different organelles and the plasma membrane, which are related to endocytosis, recycling, secretion and degradation^22,94,95^ [Fig. 3a]. RAB proteins control membrane identity and spatiotemporal dynamics of vesicle traffic through their C-terminal prenylation and interactions with effectors.^96^ RAB1, RAB22, RAB6, RAB33, RAB40, and RAB2 coordinate a series of steps during trafficking between endoplasmic reticulum (ER) and Golgi. RAB5 and RAB21 participate in regulating endocytosis^97,98^ while RAB4 and RAB11 are responsible for recycling endosomes. RAB5, RAB14, and RAB22 together mediate early formation and early-to-late maturation of phagosome,^99^ after which the late phagosome is sorted to lysosome under the coordination of RAB7.^100,101^ As such, RAB7 has a special role in targeting of late endosome, late phagosome, and autophagosome to lysosome for degradation, in which RAB24 and RAB33 regulate the formation of autophagosome together.^99,102^ RAB8 coordinates biosynthetic trafficking from Golgi to the plasma membrane and mediates insulin-stimulated translocation and fusion of GLUT4 (glucose transporter)-coated vesicles targeting the plasma membrane together with RAB10, RAB13, and RAB14.^103–106^ RAB8 also participates in regulating ciliogenesis with RAB17 and RAB23. RAB3, RAB26, RAB27, RAB29, RAB34, and RAB37 direct secretory vesicles and granules to the plasma membrane.^107–110^ RAB32 and RAB38 are involved in the biogenesis of melanosomes and RAB32 also controls mitochondrial fission.^111,112^ RAB31 is reported to drive intraluminal vesicle formation and prevent multivesicular endosome degradation during the biogenesis of exosomes.^113^ RAB18 has a still debated role in maintaining lipid droplet biogenesis and homeostasis.^114,115^ More recently, RAB22A has been shown to mediate a new type of non-canonical autophagosome formation, namely Rafeesome (RAB22A mediated non-canonical autophagosome fused with early endosome), and transfer activated STING between cells to promote anti-tumor immunity.^116^

#### Arf

Similar to RAB, Arf family proteins regulate multiple steps of vesicle transport^117^ and modulate dynamics of cytoskeleton proteins such as actin filaments and microtubules [Fig. 3b].^118,119^ Arf family is further divided into ARF, ARL (ARF-like), and SAR subfamilies. ARF1 is the best characterized ARF protein. During vesicle budding, GTP-bound ARF1 recruits coat protein-I (COPI) complex and induces positive membrane curvature for budding vesicle.^120–123^ Thus, ARF controls vesicle budding, transport between the Golgi and ER as well as transport of secretory vesicles and endosomes. Like ARF1, ARF3, ARF4, and ARF5 regulate formation of COPI-coated vesicles through ARF–COPI interactions. ARF1 also promotes the assembly of actin in the Golgi apparatus.^124^ ARF6 modulates actin polymerization and membrane traffic through colocalization with and activating PIP5-kinase, which is responsible for generation of PIP2 and directly interacts with PIP2 on the plasma membrane.^125–129^ ARLs constitute the largest subfamily of Arf family. There are 21 ARLs and they have a broad spectrum of functions, mainly involving endosome–Golgi trafficking, lysosome trafficking, cilia formation, actin remodeling and tubulin assembly. ARL2 cooperates with tubulin cofactors (TBCC, TBCD, and TBCE) in modulating αβ-tubulin assembly and microtubule dynamics.^130,131^ SAR1 has been well known for its cargo transportation from ER to Golgi.^132^ Activated SAR1 accumulates in ER, where it recruits SEC23/ SEC24 heterodimer to form a pre-budding complex. Then, the pre-budding complex binds to SEC13/31, leading to the assembly of coat protein- complex II (COPII)-coated vesicles, which are required for ER-to-Golgi cargo transport.^133^

As far as crosstalk with other small GTPases families is concerned, ARF6 binds to RhoGEF and Kalirin, recruiting them to the plasma membrane for Rac activation in the scenario of Rac-mediated cytoskeletal remodeling and membrane ruffling.^134^ TBC (Tre2–Bub2–Cdc16) domain is one key catalytic component in RabGAP proteins.^135^ Vacuole-living bacterial pathogens employ the effector proteins containing TBC-like motifs to catalyze RAB inactivation for counteracting host cell defenses, of which the binding of TBC to ARF proteins is a necessary step for implementing the GAP function.^136^

#### Ran

RAN represents a single member small GTPases family and is located close to RAB family in the phylogenetic tree. RAN plays an essential role in coordinating nucleocytoplasmic transport through the nuclear pore complex, which is involved in mitotic spindle formation^137,138^ and nuclear envelope assembly^139,140^ [Fig. 3c]. RanGTP enhances the binding of importin to the cargo and promotes the release of exportin. The asymmetric distribution of the RanGTP and RanGDP between the cytoplasm and nucleus, mediated by cytosol-localized RanGAP and nucleus-localized RanGEF respectively, generates a gradient to ensure proper transport direction of the cargo.^141,142^

#### Post-translational modifications (PTMs)

PTMs constitute an important category of mechanisms in regulating the function of small GTPases.^143^ C-terminal modifications in HVR are essential for membrane association of many small GTPases.^144^ In Ras family proteins, after synthesis is completed in ribosomes, the CAAX motif of cytosolic Ras proteins undergoes farnesylation, endoproteolysis and carboxylmethylation. The farnesylated C-terminal tail serves as the primary moiety responsible for binding to the plasma membrane.^145^ CAAX motif is located at the carboxyl-terminal, which can be farnesylated by cytosolic prenyltransferases, termed farnesyltransferase (FTase).^146,147^ FTase enhances the hydrophobicity of RAS via modifying the cysteine with 15-carbon farnesyl isoprenoid in CAAX motif covalently, and it is followed by the cleavage of AAX residues by RAS-converting enzyme (RCE1) in ER.^148^ RAS is then modified by isoprenylcysteine carboxylmethyltransferase (ICMT) and α‑carboxyl group of the farnesylated cysteine is carboxylmethylated by ICMT.^149^ A secondary modification through PTMs in the proceeding region of CaaX is needed to assist membrane attachment, which varies among different isoforms.^150^ Palmitolytion occurs in HVR of HRAS, NRAS and KRAS4A, KRAS4B, using its polybasic patch (lysine repeats) for attaching to the membrane together with the farnesylation.^150^ Farnesylation, prenylation, geranylgeranylation, and palmitolytion are four major C-terminal lipid modifications responsible for docking small GTPases to the membrane.

In G-domain of Ras proteins, PTMs have been continuously discovered within the last decade. Nitrosylation at C118 by reactive nitrogen species or oxidation by reactive oxygen species intermediates promote Ras activation via facilitating GDP-GTP exchange^151^ impeding GAP-mediated GTP hydrolysis^152,153^ while monoubiquitination at K117 in HRAS promotes its activation by rendering a fast-cycling state.^154^ K104 in KRAS is a hotspot for PTMs. However, the roles of K104 acetylation in regulating biochemical properties and oncogenicity in KRAS remain elusive.^155–157^ While the biochemical and structural properties of K104 monoubquitination in KRAS were characterized, its functional phenotype remains undetermined.^158,159^ Acetylation and methylation are two types of PTMs recently found in KRAS. Acetylation is found at multiple sites in the effector lobe and K5 methylation occurs in all different isoforms.^144^ The regulatory roles of the acetylation and methylation remain unclear and need further characterization. K147 in G5 motif is another hotspot for PTMs in KRAS, including monoubiquitination, methylation and acetylation, suggesting that potential interplay between different PTMs might provide an additional level of regulation. Phosphorylation at Y32 and Y64 by SRC reduces binding capacity of RAS proteins to downstream effectors and in particular decrease the oncogenicity of KRAS^G12^ mutants.^160–162^ The inhibitory effects by tyrosine phosphorylation can be reversed by SHP2 phosphatase, thus SHP2 has been developed as an important regulatory target for KRAS-driven cancers. Sumoylation was found at K42 in HRAS, NRAS, and KRAS and linked to the activation of RAS, but the mechanism is still undetermined.^163,164^ In a recent study, an autophosphorylation occurs at A59T mutant of HRAS and KRAS by transferring a phosphate group from the bound GTP, and the phosphorylation at T59 subsequently inhibits GTP hydrolysis and decreases binding to RAF proteins, which reveals a new mechanism by promoting nucleotide exchange and weakening the effector binding.^165^

In Rho family, phosphorylation and AMPylation are two major modifications in Switch I and Switch II regions of RhoA, Cdc42, and Rac1.^166^ Y32 in Cdc42 and Rac1, and Y34 in RhoA in Switch I region are highly conserved residues in small GTPases families and their AMPylation is found in all three isoforms, which block interactions with downstream effectors.^167^ EGF-stimulated Y64 phosphorylation in Cdc42 increases its binding to Rho GDI and is implicated in regulating the cellular localization and transformation.^168^ S71, located at the end of Switch II, is recognized as a substrate site of AKT kinase. S71 phosphorylation inhibits activation of Rac1 and Cdc42.^169,170^ In a recent study, S71 phosphorylation mediates the interaction between Rac1 and 14-3-3, an important scaffold protein involved in multiple signaling pathways.^171^ Multiple lysines in RhoA and Rac1 are involved in ubiquitination-dependent proteasome degradation, which regulates the protein expression level and impact on cytoskeleton dynamics and cell migration.^172–175^ HACE1 E3 ubiquitin-ligase, a tumor-suppressor, catalyzes ubiquitination and recruits Ubiquitin-proteasome system preferentially to the activated Rac1. This serves as a protection mechanism to ameliorate activation caused by point mutation^176^ and has also been reported to reduce reactive oxygen species generated by Rac1-dependent NADPH oxidases.^177^

Rab proteins coordinate vesicle trafficking and secretion, and the cooperation of C-terminal prenylation and regulator proteins such as Rab GDI and Rab GEF are important in sorting Rab to different subcellular locations.^178^ Phosphorylation is a major PTM that has been functionally characterized in most Rab proteins.^93^ Many kinases such as CDK, PINK1, LRRKs, PKCs, and SRC have been identified to phosphorylate different Rab proteins. Phosphorylation of Rab1 and Rab4 modulate Rab-related mitosis^179,180^ and S72 phosphorylation by TBK1 enhances the binding of Rab7 to FLCN-FNIP1, which targets damaged mitochondria during process of mitophagy.^181^ S72 phosphorylation by leucine-rich repeat kinase 1 (LRRK1) and dephosphorylation by PTEN in Rab7a dynamically regulate EGFR trafficking and degradation.^182,183^ Many Rab proteins such as Rab3, Rab8, Rab10, Rab12, Rab29, Rab35, and Rab43 can be phosphorylated by the pathological LRRK2 variants at the Thr/Ser residues located in Switch II.^184,185^ The phosphorylation by LRRK2 leads to a decreased affinity to Rab GDIs and likely alters the distribution between cytosol and membranes. Meanwhile it is also implicated in ciliogenesis through modulating interactions of RAB8A, RAB10, and RAB12 with RILPL1/2.^185^ Upon activation by mitochondrial depolarization, PTEN-induced kinase 1 (PINK1) phosphorylate S111 in Rab8A, which blocks Rabin8 (Rab8 GEF)-mediated Rab8A activation.^186^ LRRK1 and PINK1 are two important kinases in development of Parkinson’s disease (PD). As such, phosphorylation of Rabs by LRRK1 and PINK are implicated in PD progression.^187^ Other PTMs such as AMPylation, phosphocholination, and adenylylation as consequences of bacterial infection interrupt GDP-GTP cycle of Rab proteins (mainly Rab1 and Rab35 by phosphocholination), and these PTMs mainly occur at Tyr and Ser residues in switch II.^188–192^ Palmitoylation at C83 and C84 in Rab7 promotes its interactions with retromer complex and mediates endosome to trans-Golgi network trafficking of the lysosomal sorting receptors.^193,194^ K38 in Rab7A is subjected to ubiquitination by PARKIN and deubiquitination by USP32, which alters interactions of Rab7A with its effector and regulates the Rab7A-dependent endosome pathway during PD progression.^195,196^ An interesting interplay among β_2_-adrenergic receptor (β_2_-AR), HACE1 ubiquitination ligase, and Rab11A has been observed. HACE1 mediates recycling of β_2_-AR through ubiquitinating RAB11A at K145 whereas the β_2_-AR/HACE1 interactions are required for activation of HACE1.^197^ During infection by pathogenic bacterial like Legionella pneumophila, one important mechanism used by the bacteria to compromise host immune defenses is to ubiquitinate multiple RABs by the bacterial effector proteins (equivalent to E3 ligase) in an uncommon E1/E2-independent manner.^198,199^

Arf proteins have an N-terminal amphipathic helix adjacent to G-domain and myristoylation at the beginning of N-terminal is conserved in Arf proteins. Upon GDP-GTP exchange, activated Arfs release the myristoylated N-terminal to attach to the membrane.^200,201^ Acetylation at Y2 in the amphipathic N-terminal helix of ARL3, Arl14 and Arfrp1 is critical for its subcellular localizations such as recruitment to Golgi membranes.^202–204^ Methionine acetylation at N-terminal of ARL8A/B is necessary for lysosome localization and regulates lysosomal transport in cells.^205^ Palmitoylation of C8/C9 in the N-terminal of ARL13B is found to regulate protein trafficking and stability during cilia formation.^206^ Additionally, C-terminal SUMOylation of ARL13 is found to regulate ciliary targeting of sensory receptors.^207^ In comparison with other small GTPases, phosphorylation, ubiquitination, and other PTMs have been scarcely investigated for Arf proteins.^118^

Unlike other small GTPases, Ran shuffles between cytoplasmic space and nucleus rather than binding to the cellular membrane through terminal modifications. Five lysine residues (K37, K60, K71, K99, K159) located in important functional regions such as P-loop, Switch regions and G5 motif in Ran are identified with acetylation by mass spectrometry.^208^ Using unnatural amino acid incorporation strategy, a dynamic acetylation network associated with these sites has been revealed to regulate its basic GTPase activities, formation of nuclear import/export complex, and subcellular location in a site-dependent manner.^209,210^ K134 acetylation promotes Ran activation through releasing Ran from Ran-Mog1 complex and enabling RCC1 (Ran-GEF) binding. K134 acetylation in Ran has been reported to regulate the subsequent chromosome segregation in mitosis.^211^ Besides acetylation, other PTMs in Ran have been rarely characterized.

Taken together, in addition to GAP/GEF/GDI-mediated regulations, PTMs add another layer of regulation to the activity of small GTPases. In particular, many modifications occur at the switch regions. Along with technical development of proteomics, more PTMs have been found, bringing more complexity and opportunities to discover novel mechanisms in regulation and targeting of small GTPases. Many of these newly identified PTMs have been poorly characterized in terms of their effects on internal structural dynamics, GTPase cycle, molecular interactions, and relevance in diseases. There are enzymes that are responsible for installing modifications, such as transferases, kinases, E3 ligases, as well as those with roles for removing modifications including phosphatases, deacetylases, deubiquitinases, demethylases. Many of these functions are still largely unknown for most of the newly-discovered PTMs found in small GTPases. As such, the discovery of PTMs and their mechanistic characterizations has created an exciting time in the research of small GTPases.

## Small GTPases in human diseases

### Ras

**Fig. 4:**
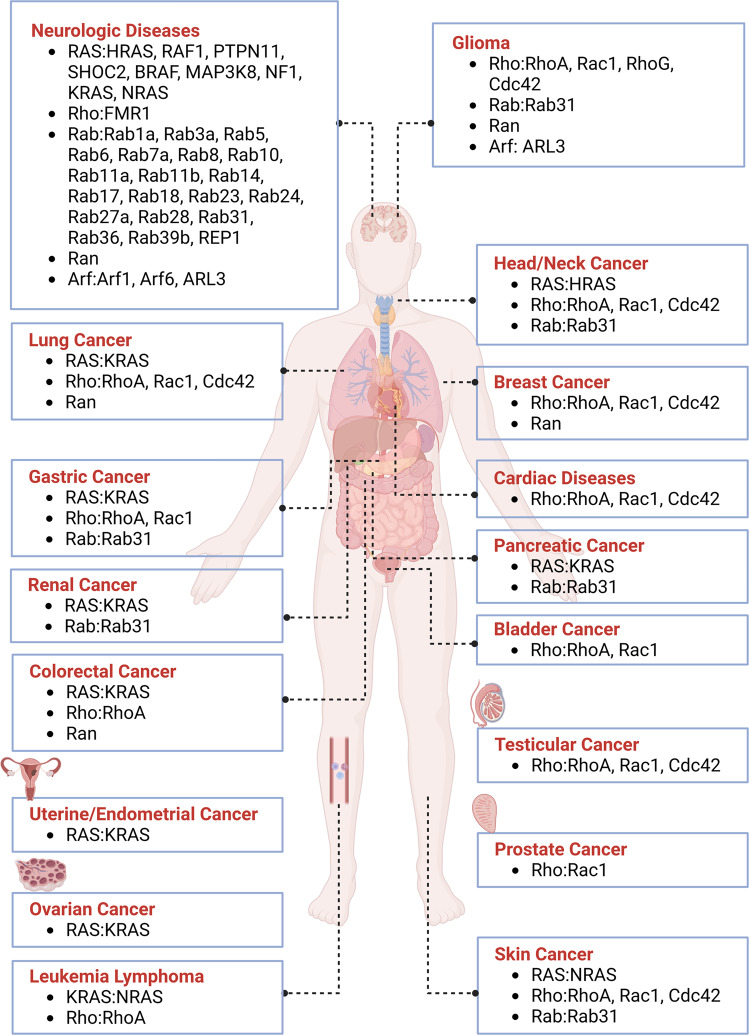
Small GTPases in human diseases. Distribution of mutations or aberrant expression of small GTPases across human diseases including cancers and neurologic diseases. This figure was created with BioRender.com

Ras family proteins are associated with a wide range of human diseases (Table 1). Somatic KRAS mutations are present in many different human cancers, including pancreatic adenocarcinoma, colorectal cancer, non-small cell lung cancer, cholangiocarcinoma, multiple myeloma, uterine cancer, endometrial cancer, gastric cancer, testicular cancer, cervical adenocarcinoma, diffuse large B-cell lymphoma, breast cancer, acute myeloid leukemia, chronic lymphocytic leukemia, bladder cancer, and cutaneous malignant melanoma^212^ [Fig. 4]. Mutations of other isotypes, NRAS and HRAS are found in many human cancers as well. For instance, NRAS Q61L is one of dominant drivers of melanoma. The most commonly mutated residues in Ras proteins include G12, G13, and Q61, as these mutants impede GTP hydrolysis and lead to constitutive activation of Ras.^213^ In addition to tumors, RASopathies are a group of genetic disorders caused by germline mutations in genes that encode proteins of RAS/MAPK pathway and their regulatory proteins.^214–216^ The developmental disorders characterized as RASopathies include neurofibromatosis, Noonan syndrome, and Costello syndrome.^217^ These conditions share common clinical features such as facial abnormalities, cognitive impairment, and congenital heart defects, due to aberrant activations of RAS/MAPK pathway.^218^ Furthermore, abnormalities in the RAS-MAPK pathway have been found in nearly 80% of therapy-relapsed neuroblastoma samples, which indicates that personalized therapy targeting specific RAS mutations as well as a sensitivity to MEK inhibition may be effective in the treatment of neuroblastoma.^219,220^ While neuroblastoma was initially believed to be largely attributed to other cellular mechanisms such as activating ALK or inactivating ATRX mutations, refractory neuroblastoma cases have been shown to express mutant Ras proteins, most commonly NRAS Q61K and HRAS Q61K.^220^ Furthermore, activation and increased expression of RALs are observed in many RAS-driven tumors.^221^ RALs are further shown to contribute to anchorage-independent cell growth and invasion of multiple cancers such as lung cancer, colorectal cancer, melanoma, pancreatic cancer and bladder cancer;^59,221,222^ RALA is required for anchorage-independent growth while RALB is necessary for the survival of tumor cells.^223^ Increased expression and activation of both RALs are associated with enhanced tumor growth. Owing to its broad impacts in many cellular processes, RASA1 associated disorders are characterized by capillary and arteriovenous malformations, which contribute to heart failure in affected patients.^224^

**Table 1:** Small GTPases in human diseases

Small GTPases Family	Disease Related to Family Dysfunction	Common Implicated Mutations/Dysregulations
Ras	Pancreatic adenocarcinoma,^212^ colorectal cancer,^212^ non-small cell lung cancer, cholangiocarcinoma,^212^ uterine endometrial cancer,^212,541,542^ neuroblastoma^219,220^	*KRAS* ^*G12, G13, Q61*^
	Malignant melanoma,^212^ leukemia,^212^ lymphoma^212^	*NRAS* ^*G12, G13, Q61*^
	Costello syndrome,^217^ salivary gland cancer^543^	*KRAS* ^*G12, G13, Q61*^
	Noonan syndrome^217^	*KRAS*, *NRAS*, *RAF1*, *PTPN11*, *SHOC2*, *BRAF*, *MAP3K8*
	Neurofibromatosis^217^	*NF1*
Rho	Burkitt lymphoma,^225,544^ T-cell lymphoma,^225,545–547^ Kaposi’s sarcoma,^548^ Alzheimer’s disease,^20^ Huntington’s disease^19^	*RHOA1*
	Pulmonary hypertension,^234^ urothelial carcinoma,^549^ psoriasis,^232^ epilepsy,^550^ prostate cancer^551^	*RAC1*
	Rheumatoid arthritis,^236^ systemic sclerosis,^237^ testicular cancer,^552^ gastric cell carcinoma^553^	*RHOA1*, *RAC1*
	Breast cancer,^554,555^ non-small cell lung cancer,^556,557^ squamous cell carcinoma^558,559^	*RAC1*, *CDC42*
	Atherosclerosis,^21,235^ angina,^560^ myocardial infarction,^561^ aortic aneurysm,^562^ cardiac hypertrophy,^563,564^ Parkinson’s disease^336^	*RHOA1*, *RAC1*, *CDC42*
	Glioblastoma^565–567^	*RHOA1*, *RAC1*, *RHOG*, CDC42
	Melanoma,^568,569^ squamous cell carcinoma^570^	*RAC1* ^*P29S*^
	Neutrophil immunodeficiency^571^	*RAC2* ^*D57N*^
Rab	Charcot-Marie-Tooth disease^572^	*RAB7* ^*L129, K157, N161, V162*^
	Warburg Micro syndrome^573^	*RAB18*
	Carpenter syndrome^245^	*RAB23*
	Griscelli syndrome^246^	*RAB27A*
	X-Linked intellectual disability^574^	*RAB39B*
	Choroideremia^575^	*REP1*
	Ovarian cancer,^24^ breast cancer,^24,576^ renal cancer,^577,578^ gastric cancer,^579^ liver cancer,^580^ non-small cell lung cancer,^581^ bladder cancer,^582^ glioblastoma multiforme,^583^ prostate cancer^584^	*RAB25*
	Glioblastoma,^585^ pancreatic cancer,^586^ renal cell carcinoma,^587^ oral squamous cell carcinoma,^588^ breast cancer^589^ and gastric cancers^590^	*RAB31*
	Alzheimer’s disease^238^	*RAB1A*, *RAB3A*, *RAB5*, *RAB6*, *RAB7*, *RAB8*, *RAB10*, *RAB11A*, *RAB11B*, *RAB14, RAB17*, *RAB24*, *RAB27*, *RAB36*
	Cone-rod dystrophy^242^	*RAB28*
	Parkinson’s disease^187,238^	*RAB1*, *RAB7*, *RAB11*, *RAB39B*
Ran	Colorectal cancer,^250^ frontotemporal dementia,^591^ glioblastoma,^592,593^ Alzheimer’s disease,^36^ breast cancer,^362,594^ lung cancer^595,596^	*RAN*
Arf	Creutzfeld-Jakob disease^597^	*ARF1*
	Periventricular heterotopia,^598^ retinitis pigmentosa, diabetic kidney disease, amyotrophic lateral sclerosis^599^	*ARF6*
	Gliomas^254^	*ARL3*
	Meckel-Gruber syndrome,^600^ Joubert syndrome,^600^ nephronophthisis^601^	*ARL13B*
	Bardet-Biedl syndrome^259,260^	*ARL6*
	Chylomicron retention disease^261^	*SAR1B*

### Rho

The Rho family affects a wide variety of cellular functions and thus contributes to many human pathologies [Fig. 4], (Table 1). Primarily, Rho, Cdc42, and Rac proteins are associated with tumorigenesis and cardiovascular disease, including lymphomas, Kaposi’s sarcoma, prostate cancer, gastric cell carcinoma, breast cancer, non-small cell lung cancer, squamous cell carcinoma, and glioblastoma. Movement of tumor cells is important in tumor progression and processes including formation and extension of pseudopods, establishment of new adhesion sites, contraction of cell bodies, and retraction of the tails are regulated by many different mechanisms.^225^ Rho subfamily is one of the key regulators in cell-matrix adhesion and cytoskeletal reorganization, thus regulating the invasion of tumor cells.^225^ Cdc42 communicates with the RAS subfamily to induce RAS-mediated transformation into carcinogenic cells through cell cycle progression.^226^ Additionally, Rac1 has been linked to the development of atherosclerosis and vascular disease. Rac1 is essential in the production of reactive oxygen species by NADPH oxidase as well as the migration of endothelial cells of blood vessels during shear stress, two processes that are vital contributors to atherosclerosis.^21^ The formation of reactive oxygen species following cardiac myocyte injury is one of the key processes in the development of cardiac hypertrophy. In one study, DL0805 has been shown to be a possible therapeutic agent in cardiovascular disease by acting as a vasorelaxant in rat thoracic aortas through inhibition of the Rho/ROCK signaling pathway.^227^ RhoA is required for the phosphorylation of myosin light chain, a key event in the regulation of vascular smooth muscle cell contraction. Rho-kinase and nitric oxide have been shown to have opposite effects on lipid metabolism. Nitric oxide activates hepatic sterol regulatory element-binding protein-2 which is a transcriptional factor for cholesterol metabolism and the expression of LDL receptors, decreasing the burden of cardiovascular disease. RhoA suppresses whole body energy consumption by inhibiting AMPK, thus contributing to dyslipidemia.^228^ As such, Rho plays a crucial rule in the pathogenesis of coronary artery vasospasm, which can cause angina and myocardial infarction. In the central nervous system, Rho acts by regulating axonogenesis, neuronal migration, and synaptic plasticity, and mutations in Rho proteins contribute to several neurologic disorders. Increased level of Rho was found in postmortem brains of patients with Huntington’s disease and Alzheimer’s disease.^19,20^ Cdc42 G12V and Q61L mutations, found in the same positions as RAS hotspot mutations, result in constitutively active proteins that exhibit oncogenic activities.^229^ Cdc42 is especially important in migration of tumor cells. In thyroid cancer, overexpression of Cdc42 increases production of lactic acid and polarization of M2 macrophages, which function to inhibit T cells and allow cancer cells to proliferate uninhibited.^230^ Cdc42 is also thought to have an essential role in the regulation of epileptic seizures, as pretreatment with ML141, a Cdc42 inhibitor, was found to reduce seizure severity.^231^ Finally, in the skin, constitutively active Rac1 (RACV12) in mice resulted in the development of lesions similar to human psoriasis and inhibition prevented psoriasis hyperplasia in xenografts. Rac1 modulation was shown to affect epidermis-immune reactions in inflammatory pathways including STAT3, NFKB, and zinc finger protein 750,^232^ contributing to the pathophysiology of melanoma, squamous cell carcinoma, and neutrophil immunodeficiency. Additionally, Rac1 is commonly hyper-expressed in some types of cancer, including urothelial carcinoma. Inhibition of Rac1 has been shown to prevent metastasis of bladder cancer.^233^ In non-cancerous pathology, Rac1 contributes to pulmonary hypertension via its role in NO-mediated smooth muscle relaxation.^234^ Disruption of Rac1 macrophage regulation has been shown to increase disease stability in atherosclerosis as well,^235^ highlighting its role in development of the disease. RhoA and Rac1 have been shown to be crucial in the development of some rheumatologic diseases as well, including rheumatoid arthritis^236^ and systemic sclerosis.^237^

### Rab

Dysregulation in the Rab family is implicated in human diseases related to disrupted intracellular membrane trafficking and vesicle movement [Fig. 4], (Table 1). Rab is predominantly expressed in the brain and thus has a large effect on the development of neurologic diseases, such as neurodegenerations including PD and AD.^238^ PD is a disease largely caused by death of neuronal cells due to accumulation of misfolded proteins in the substantia nigra. Rab29a and Rab39 have been shown to play important roles in the development of PD, as inactivating mutations in these proteins prevent proper expression of surface receptors, such as AMPA, required for proper secretory trafficking.^30^ In AD, aberrant expression of Rab5 results in abnormally large early endosomes due to dysfunctional membrane trafficking. In mice, Rab5 overexpression was found to induce AD-like neurons and pathology that was previously attributed to AB/Beta amyloid, signifying that Rab5 may be a worthwhile therapeutic target for AD.^31^ Aberrant expression of Rab25 and Rab31 have been implicated in development of multiple types of cancer.^239,240^ Rab35 mutations can activate PI3K and AKT in tumor progression.^241^ Dysfunction in the Rab pathway has also been known to contribute to hearing loss and cone-rod dystrophy.^242^ Gain of function mutations in Rab7 have been linked to the development of Charcot-Marie-Tooth Type 2B disease.^243^ Furthermore, Rab mutations have been implicated in other immune disorders,^244^ such as Carpenter syndrome (RAB23),^245^ Griscelli syndrome (RAB27A),^246^ and Hermansky-Pudlak syndrome (RAB38).^247,248^

### Ran

Ran is overexpressed in breast and lung cancers and knockdown of Ran leads to a reduction of Met receptor expression that contributes drug resistance of trastuzumab and gefitinib^249^ [Fig. 4], (Table 1). Ran expression is upregulated in metastatic colorectal cancers^250^ and downregulation of Ran increases senescence in normal cells. The activation of Ran is critical for the activity of nuclear-cytoplasmic transport. Increased level of Ran and thus nuclear-cytoplasmic transport allow the cell to evade DNA damage-induced cell cycle arrest and senescence, which is vital for cancer cells.^251^ In neurologic conditions, reduced expression of Ran was observed in AD^36^ [Fig. 3]. It also plays a crucial role in frontotemporal lobar degeneration by regulating TDP-43 induced retinal degeneration.^36^

### Arf

The Arf family plays key roles in tumor progression and invasion, in particular tumor angiogenesis^252^ [Fig. 4], (Table 1). During neurological development, Arf6 is highly expressed in the human brain and Arf6 dysfunction leads to defects in the cellular migration that contribute to neuronal disease.^253^ Defective Arf-related processes are involved in autosomal recessive periventricular heterotopia, retinitis pigmentosa, and amyotrophic lateral sclerosis as a consequence of defective neuron migrations. ARL3 is implicated in pathogenesis of gliomas. Low expression of ARL3 in gliomas predicted poor prognosis, likely due to its essential role in angiogenesis and immune cell infiltration in the microenvironment of the cancer.^254^ Arf6 is involved in the accumulation of cholesterol in podocytes in diabetic kidney disease.^255^ Legionella pneumophila and Rickettsia prowazekii, the bacteria responsible for Legionnaire’s disease and epidemic typhus respectively, utilize a Type IV secretion system to infect target cells. One of these effector molecules is RalF, which contains a domain homologous with Arf GEFs.^256^ The bacteria utilize this homology to recruit Arf to bacteria-containing vacuoles and allow the bacteria to proliferate.^257^ Furthermore, ARL13B mutations have been shown to contribute to the pathophysiology of inherited disorders such as Meckel-Gruber syndrome, Joubert syndrome, and nephronophthisis. ARL13B is found to contribute to the structure of the ciliary membrane and its defects prevent cellular transport and diffusion across the membrane from occurring properly.^258^ ARL6 mutants with disrupted nucleotide binding are reported to cause ciliary transport defects in Bardet-Biedl syndrome.^259,260^ Finally, SAR1B is a member of the Arf family which plays a key role in secretions of chylomicrons into the small intestine, and mutations in this protein result in Chylomicron retention disease.^261^

## Targeting small GTPases in human diseases

Due to their significant prevalence in human diseases, there has been a long journey for developing strategies to target small GTPases. Among them, Ras family proteins such as KRAS have been established as a paradigm for targeting Ras proteins and other small GTPases. Since the first KRAS^G12C^ inhibitor (Sotorasib or AMG510) has been approved by the FDA in November 2021,^262,263^ many new targeting strategies have been discovered during the last decade through concerted efforts of chemical biology, structural biology, and fragment-based drug discovery (FBDD). In this section, we will review the progress made in the field of targeting Ras and other small GTPases with an emphasis on emerging strategies. We will divide the topic into two major categories: direct targeting and indirect interventions. Because of the similarity in the structure and basic biological function shared between different small GTPases families, we expect this summary will booster drug discovery progress for other small GTPases through dissemination of the methods developed for KRAS.

### Direct targeting

Assuming that GTP and ATP share similar chemical structures and most of the kinase inhibitors are ATP analogs, the early attempts to inhibit Ras were focused on developing molecules to compete with GTP. However, this has been in vain because the picomolar affinity between GTP/GDP and Ras proteins is untamable. Moreover, the lack of effective pockets at the protein surface poses another tremendous challenge for applying the traditional drug development methods to target Ras proteins. As such, the progress for targeting Ras proteins has been stalled for decades and Ras has been notoriously described as “undruggable”. More recently, the field has regained new momentum as the pockets associated with the switch regions have been successfully exploited for small molecule binding as well as the surfaces at the effector lobe for macromolecular inhibitor binding. Most of the inhibitors are designated to interrupt the GEF-mediated Ras activation process (GDP-GTP exchange) or interfere with effector binding. Until very recently, mutation-specific covalent binding strategies (PROTACs, CLAMPs, etc.) have been developed for targeting KRAS, which serve as proof of concepts and technical repertoire for targeting other Ras proteins and small GTPases. Therefore, the dawn of the “druggable” is rising.

#### Exploiting structural plasticity of Switch regions

Direct targeting relies on exploring structural motifs at protein surfaces for accommodating ligand binding with structural specificity. In the last decade, many milestones have been reached in targeting KRAS by diversified efforts of structure-based drug discovery. Most of the ligand binding pockets are associated to switch regions of KRAS due to their significant structural plasticity. Their conformations and dynamic properties are tightly modulated by GDP/GTP binding or interactions with regulatory or effector proteins [Fig. 1f, g]. As a proof of the conformational selection theory, a recent NMR study reveals that many reported ligand binding pockets co-exist as minor conformations in dynamic assembles mainly comprised of switch regions in the KRAS unbound to ligand,^49^ indicating that the structural plasticity and dynamics are the key factors to guide structure-based drug screen and design for KRAS and other small GTPases. According to the location, there are two major cavities for ligand binding, Switch I/II pocket (a pocket between Switch I and Switch II) and Switch II pocket.

#### Switch I/II pocket (SI/II-P)

**Fig. 5:**
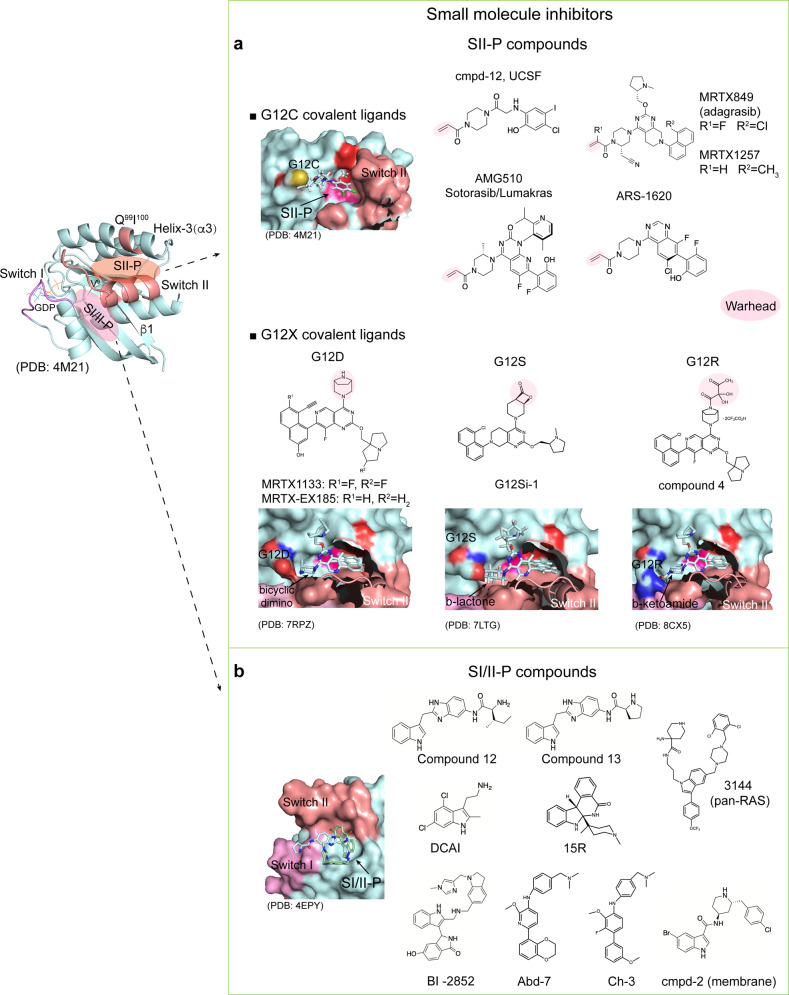
Direct targeting of small GTPases by small molecules. **a** G12C-specific inhibitors bind to SII-P with the covalent warheads, other ligands specifically bind to SII-P of KRAS hotspot mutants (G12D/S/R). **b** Compounds bind to SI/II-P of WT KRAS and different mutants

Switch I/II pocket is a shallow and small pocket located between Switch I and Switch II [Fig. 5b], previously tagged as “undruggable”. This stigma has been removed by advancements of FBDD efforts over last decade. A key characteristic of the FBDD approach is to pick up small molecules with low molecular weight (<300 kDa) and weak affinity but high potential for optimizations and expansion in chemical space. When applying FBDD to Ras proteins, two structure-based methods, nuclear magnetic resonance (NMR) and virtual screen, play important roles in finding fragment hits binding to SI/II-P. In 2012, DCAI compound (Kd: ~1.5 mM, Genentech) and compound 12 (Kd: 190 μM, Vanderbilt) were discovered by NMR-based FBDD. Both of these compounds bind to Switch I/II pocket and block Ras-SOS (RasGEF) interactions, which are required for Ras activation.^264,265^ A year later, Kobe2602, a compound derived from a virtual screening, was shown to bind to a similar pocket in HRAS^T35S^ mutant and reduce Ras-Raf binding and downstream signaling.^266^ Following this path, a fragment screening conducted on active KRAS (bound to GMPPCP) by NMR led to the identification of BI-2852, which binds to SI/II-P at nanomolar affinity (*K*_D_).^267^ BI-2852 binds to both inactive and active KRAS, diminishes protein–protein interactions (PPIs) between KRAS and GEF/GAP/effectors, and demonstrates unambiguous dose-dependent reduction in phosphorylation level of ERK and MEK kinase with antiproliferation effects in cell models.

NMR is evidently instrumental in FBDD efforts to target SI/II-P with respect to its advantages in screening, structure mapping, hit validation, and optimization. Two recent articles have reviewed the progress made on structural and dynamic characterizations and drug discovery for small GTPases by NMR.^268,269^ Other strategies have also been employed to develop SI/II-P binders. A comparative screening conducted between Ras complexed with an intracellular antibody fragment (i.e., anti-Ras VH chain) and Ras alone can elucidate the compound that binds to the VH epitope. The compound Abd-7 binds to SI/II-P, interrupts interactions of KRAS with its effector proteins, and reduces its downstream signaling.^270^ The Abd compounds were further optimized by fusing with the functional groups screened by the crystal soaking method and the fused compound, ch-3, exhibits enhanced potency.^271^ Besides this, a pan-Ras inhibitor (3144) from virtual screening has been found to interact with different sites located at SI/II-P and displays anti-tumor activity in a xenograft mouse model and the human breast cancer cell line (MDA-MB-231) that carry KRAS G13D mutation.^272^ Natural products constitute another important resource enriched with naturally occurring molecular architectures to be exploited for drug discovery. Tricyclic indolopyrrole alkaloid and indoloisoquinolinones (15R) were identified from a natural products screen and structurally verified to bind to SI/II-P of GMPPCP-bound KRAS^G12D^.^273^

#### Switch II pocket (SII-P)

SII-P consists of Switch II, helix-3 (H3), and the end of β-sheet 1 (β1). It is a well-known pocket [Fig. 5a] for G12C inhibitors including Sotorasib. The earliest effort to screen ligand binding to this pocket by NMR was in 1997, which led to the identification of a sugar derivative ligand, SCH-54292.^274,275^ Even with limited affinity, the discovery of SCH-54292 provides an alternative possibility to target the allosteric site instead of directly outcompeting GDP or GTP. In 2013, a series of high-affinity SII-P ligands have been developed by taking advantage of endogenous reactivity from the cysteine sidechain of oncogenic KRAS^G12C^ mutant, an oncogenic mutation presenting in 13% NSCLC patients in the United States.^276^ A combinatory approach was applied by integrating two moieties containing a lead binding to SII-P and an electrophilic warhead covalently attaching to the thiol group of the cysteine.^276^ This serves as a milestone to target KRAS and opens a new avenue that drives the potent G12C inhibitors approved by FDA or under evaluations of clinical trials, such as Sotorasib from Amgen,^262,263^ Adagrasib (MRTX-849) from Mirati, and JNJ-74699157 (ARS-3248, an updated version of ARS 1620) from J&J.^277–281^ Medicinal chemistry plays a key role in evolving compounds with greater potency and efficacy by optimizing chemical spaces for both the SII-P binding moiety and warhead. It has been proven that binding of G12C SII-P covalent inhibitors reshapes conformation of switch regions to favor GDP-binding by abrogating the interactions between γ-phosphate and Switch II region.^282^ As such, the KRAS mutant is retained in the inactive “*off*” state and the *KRAS*^*G12C*^-driven tumor growth is inhibited.

G12C only represents ~12% of KRAS mutations in human cancer according to the statistics from COSMIC database, but targeting other oncogenic KRAS mutations remains challenging. Inspired by the successes of the covalent G12C ligands, covalent ligands for G12S and G12R mutants have been recently developed. In the G12S ligand, an electrophilic group, β-lactone, specifically reacts with a serine residue and has been attached to the tetrahydropyridopyrimidine moiety of the G12C ligand Adagrasib.^283^ The G12S covalent ligands, G12Si-1/5 exhibit inhibitory effects to both KRAS GTP-loading and phosphorylation of downstream ERK in tumor cell lines containing KRAS G12S mutation. Similarly, a compound that contains an α, β-diketoamide warhead (compound-4) selectively binds to GDP-bound G12R mutant through covalent binding to the sidechain nitrogen of arginine 12. In contrary to the G12C mutant, the G12R mutation leads to severe loss of GTP hydrolysis activity and dominantly exists in the GTP-bound state, thus the current version of the G12R covalent ligand shows limited activity in cells and further optimization is needed.^284^

G12D is the most prevalent KRAS mutation in pancreatic cancer and colorectal cancer, however, the aspartic acid mutation does not render the same chemical reactivity as the cysteine mutation. Through chemical space optimization based on the G12C inhibitors, a noncovalent KRAS^G12D^ inhibitor, MRTX1133, has been recently reported with good selectivity and potency.^39,285^ Starting from the established SAR of the G12C inhibitor Adagrasib, a pyrido[4,3-d] pyrimidine scaffold was chosen and a substitution of the reactive warhead with [3.2.1] bicyclic diamino substituent at the C4 position was made for selectively interacting with the negatively charged sidechain and the neighboring residues in the P-loop. Then, further optimizations were made with a substituent 2-fluoro-pyrrolizidine at the C2 position and with a substituent 7-fluoro-8-ethynylnaphthyl at the C7 position in order to sterically fit better into SII-P of G12D mutant. The final product termed MRTX1133 displays a sub-picomolar binding affinity in a non-covalent manner. Furthermore, MRTX1133 induces tumor regression to most of the PDAC tumor models through effective inhibition of G12D signaling.^38,286^

Presently, there are inhibitors targeting different alleles of KRAS including G12C, G12D, G12S, and G12R under development and KRAS is evidently becoming *druggable*. One key question is whether SII-P is the only accessible pocket to the covalent ligands and the GDP-loaded protein. In a new study, the G12C covalent ligands Adagrasib, MRTX1257, and G12D ligand MRTX-EX185 have been found to reversibly interact with SII-P of GDP-bound WT KRAS, KRAS^G12D^, and WT HRAS. MRTX-EX185 also binds to GTP-bound form of all three.^287,288^ More interestingly, all three MRTX compounds exhibit in-cell target engagement across WT KRAS and the hotspot mutants including G12C, G12D, G13D, Q61H, and Q61L. Furthermore, MRTX-EX185 engages G12V and Q61R mutants in cells. Unlike MRTX compounds, Sotorasib exclusively interacts with the G12C mutant. Both MRTXs and Sotorasib bind to SII-P but the residues involved in the interactions are partially different, which led to the emergence of different secondary KRAS mutations in relapsed tumors following treatment with different drugs.^289^ Taken together, SII-P widely exists across Ras isoforms and KRAS hotspot mutants as well as both GDP- and GTP-bound forms, confirming that the structure of SII-P and dynamic modulations of switch region conformations in different hotspot mutants is important for discovery of novel mutant-specific ligands to address the stringent needs of precision medicine. Furthermore, the strategies and experimental methods evolved during the last decade for targeting SII-P as well as SI/II-P of KRAS shed light on drug discovery for other Ras isoforms and small GTPases.

In addition to target the SII-P, the progress achieved with G12C covalent inhibitors also revived the concept of competing with GDP/GTP in binding to the guanine nucleotide-binding pocket. Compound SML-8-73-1 represents another type of G12C covalent inhibitor by binding to the nucleotide pocket and rendering KRAS locked in an inactive conformation with reduced cellular activity.^290,291^ Ras-like proteins (RALA and RALB) are located at Ras branch in the phylogenetic tree of small GTPases and share high structural similarity with Ras proteins. Interestingly, Rals are also located at a downstream signaling node of the Ras pathway (Ras-RalGDS-Ral). Activation and upregulation of Rals are observed in many Ras-driven tumors, indicating the potential of Rals as cooperative targets with Ras for cancer treatments.^221^ In a parallel study for Ral inhibitors, two compounds, RBC8 and BQU57 from virtual screen, were found to bind to a pocket mainly composed by residues in Switch II with ~100 μM affinity. They inhibit tumor growth in lung cancer xenograft models.^292^

#### Macromolecular inhibitors

Therapeutic macromolecules such as peptide ligands, proteins, and antibodies represent an alternative Ras targeting approach. Compared with small molecule ligands, macromolecular ligands exhibit improved selectivity and binding capacity to compete with endogenous PPI partners such as GEFs and effectors.^293^

#### Cyclic peptide ligands

**Fig. 6:**
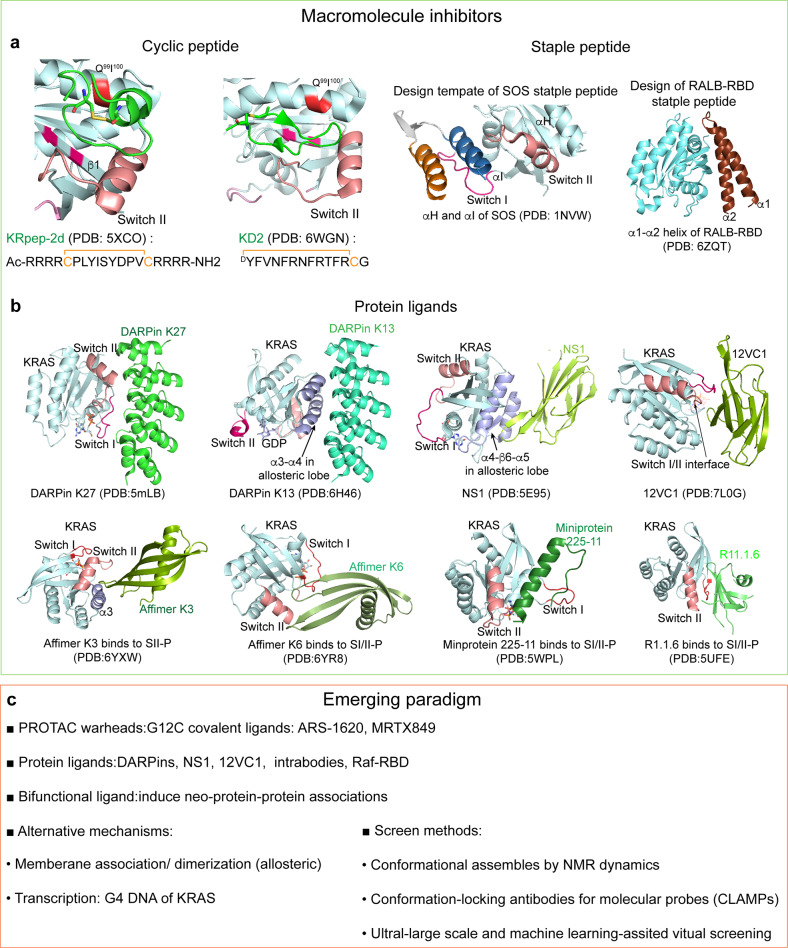
Direct targeting of small GTPases by macromolecules and emerging paradigm. **a** Cyclic peptides and staple peptides bind to KRAS and RALB. **b** Protein ligands interact with switch regions or allosteric lobe of KRAS. **c** Newly-developed targeting strategies and drug screening methods

A cyclic peptide screened from bacteriophage display binds to part of SII-P of KRAS^G12D^ mutant with 10 nm affinity (*K*_D_) and six-fold selectivity over WT KRAS [Fig. 6a]. It disrupts Ras activation and effector binding to SII-P in vitro and inhibits proliferation in G12D mutant cancer cell models and KRAS^G12D^-driven tumor growth in animal models.^294–296^ In another study, a bicyclic peptidyl pan-Ras inhibitor, cyclorasin B4-27, binds to GTP-bound form of WT KRAS and G12V mutant with ~20 nm affinity (*K*_D_). NMR characterizations indicate the binding site of B4-27 is mainly overlapped with SI/II-P.^297,298^ With continuous improvement in chemical compositions, cellular permeability, and metabolic stability, B4-27 displays good potency in blocking Ras-effector interactions and induces apoptosis of multiple cancer cell lines containing KRAS or HRAS mutants. A 16-mer cyclic peptide, screened from a naïve library, binds to non-switch-region of Cdc42 at nanomolar affinity and inhibits RAS-driven tumor cell proliferation and invasion in a Cdc42-dependent manner.^299^

Through an integrative approach of mRNA display, in vitro translation, and unnatural amino acid substitution, a cyclic peptide, KD2, was identified to preferably bind to GTP-bound KRAS^G12D^ mutant at a groove mainly involving SII-P region and block the interactions of KRAS with CRAF RBD.^300^ Its selectivity to the mutation allele and nucleotide-bound form presents a promising approach to target the challenging oncogenic mutant.

#### Staple peptides

Staple peptides [Fig. 6a] are capturing increased interests in developing peptide inhibitors for KRAS and other small GTPases. The design of staple peptides harnesses the potentials of binding affinity and selectivity inherited from the peptide motifs located at the PPI interface. In the early attempt, a helical peptide derived from a crucial helical Ras-binding motif (αH: F929-N944) at SOS surface was designed with hydrogen bond surrogate (HBS) strategy to stabilize the helical conformation. The HBS helical peptide binds to a surface spanning both Switch I and II at Ras with an affinity (*K*_D_) ~ 100 μM.^301^ By engineering the helical motif with hydrocarbon insertions, the helical peptide ligand binds to Ras with nanomolar affinity by fitting into SI/II-P and blocks guanine nucleotide binding.^302^ The hydrocarbon helical peptide demonstrates inhibitory effects in cancer cell models containing KRAS G12 or G13 mutations and Drosophila melanogaster model corresponding to G12V mutation. In a recent study, αH and another helical motif (αH: 960–975) from Ras-interaction interface of SOS are engineered together via a synthetic linker, termed as CHD^Sos^-5.^303^ As an extended staple peptide or proteomimic, biophysical characterizations reveal CHD^Sos^-5 can bind to both switch regions in nucleotide bound form in contrast to preferable binding of HBS to the nucleotide-free Ras. CHD^Sos^-5 resists proteolysis, enters cells through micropinocytosis, and exerts inhibitory effects by suppressing downstream signaling in cancer cells with high levels of micropinocytosis, such as T24 (*HRAS*^*G12V*^) bladder and H358 (*KRAS*^*G12C*^) lung cancer cells. CHD^Sos^-5 binds WT HRAS with six to eight folds higher affinity to G12V/D/C mutant. Moreover, it interacts with KRAS, RAN, and multiple Rabs according to chemoproteomic analysis, suggesting that potential off-target effects be taken into account. Screened from a naïve library, a “mini-protein” containing two helical components and a loop linker shows picomolar binding affinity to the switch regions of KRAS with an interesting observation that the interactions induce formation of a groove connecting SI/II-P and SII-P.^304^ Similar to Ras, staple peptides derived from RLIP76 RBD, a Ral effector, selectively binds to Rals with affinity at single digit micromolar (*K*_D_) and interrupts the PPIs.^305^

Peptide drugs including cycling peptides and staple peptides demonstrate many advantages like high affinity to compete with the endogenous PPIs of small GTPases. As such, the effective inhibition is achieved through interrupting interactions with GEFs or effectors, which are difficult for small molecules. A major limitation for therapeutic peptides is their short plasma circulation time because the natural peptide bonds are prone to enzymatic cleavages. Other limitations include low oral bioavailability and cell penetration. To address these issues, strategies such as chemical modifications, incorporation of unnatural amino acids, adjusting molecular size, conjugations to other functional molecules or peptides have been employed.^306^ For peptide ligands targeting small GTPases, further developments to improve selectivity towards different subfamily isoforms or disease mutants are also needed.

#### Protein ligands

Protein ligands [Fig. 6b] developed for targeting Ras proteins within the last decade mainly include antibody mimetics/fragments, monobody, and other interacting proteins. The prototypical immunoglobulin antibody contains multiple disulfide bonds that are volatile in the reducing environment of cytosol. A cysteine-free antibody mimetic with low molecular weight (<20 KDa), DARPin K27, was obtained from phage display, binds to a surface spanning both Switch regions of KRAS with 4 nm affinity (*K*_D_) and exhibits potent intracellular inhibitions to Ras downstream signaling.^307^ Other DARPins, K13 and K19, bind to helix α3-loop 7-α4 in the allosteric lobe of KRAS and inhibit downstream signaling through interruption of GEF-mediated activation, dimerization, and possible allosteric perturbations on conformation of switch regions.^308^ NS1 monobody, a synthetic binding protein, binds to α4-β6-α5 region in the allosteric lobe of KRAS and HRAS, but not NRAS. It inhibits RAS interaction with RAF kinase by disrupting Ras dimerization at the membrane surface.^309^ In another study, a monobody, 12VC1, exhibits up to 400-fold selectivity for binding to GTP-bound G12V and G12C mutants compared to WT KRAS. It suppresses ERK phosphorylation and KRAS-driven cancer cell proliferation in vitro and tumor growth in vivo.^310^ In contrary to NS1 monobody, 12VC1 binds to an interface composing of switch I and switch II. Besides this, intrabody, the cysteine-less antibody fragments in low molecular weight, have been developed to target Ras proteins.^311–314^ Switch pockets of KRAS are also major targets for protein-ligand binding: Affimer K3 binds to SII-P, Affimer K6 binds to SI/II-P,^315^ miniprotein 225–11 binds to SI/II-P with midpicomolar affinity^304^ and R11.1.6 binds to SI/II-P with nanomolar affinity.^316^ JAM20, another monobody, binds to SI/II-P of all Ras isoforms with nanomolar affinity at both GDP- and GTP-bound states.^317^ It competes with RAF-RBD, thereby effectively inhibiting the Ras downstream signaling and Ras-driven transformation in vitro and in vivo.

Applications of macromolecules to target intracellular proteins remain clinically limited due to the practical difficulty in entering cells. Nevertheless, the cases of DARPins, NS1, and 12VC1 point out that interactions occurring at regions in the allosteric lobe and switch I/II modulate Ras functions through distinct mechanisms. These binding proteins can also be used as probes to find small molecules that selectively bind to the allosteric sites. Furthermore, the effector lobe (a.a. 1−86) in Ras is strictly conserved, while the allosteric lobe (a.a. 87–166) determines the differences among different Ras isoforms. As such, targeting the allosteric lobe can be an alternative strategy to develop isoform-specific Ras inhibitors.

#### Emerging paradigms

Many cutting-edge targeting approaches [Fig. 6c] are evolving on the basis of chemical biology, medicinal chemistry, and structural biology, such as targeted degradations by PROTACs. Other attractive platforms have also been developed for targeting Ras, including molecular glues and CLAMPs. Regulation mechanisms such as membrane associations and genetic modulations, as well as targeting the allosteric lobe, offer potential new angles for RAS inhibition.

#### Targeted degradations

It is not surprising that KRAS is an attractive battlefield for method developments and applications of PROTACs. ARS-1620 and Adagrasib, established as G12C covalent inhibitors, were integrated with E3-ligase binders to develop PROTAC degraders for targeting KRAS^G12C^ mutant. The KRAS^G12C^ PROTAC degraders demonstrate potent inhibition of KRAS downstream signaling and antiproliferation effects.^318,319^ Following the path of protein ligands, DARPins, NS1, 12VC1, intrabodies and Raf-RBD were fused to functional domain of E3 ligase respectively for the targeted degradation of RAS.^310,320,321^ These engineered protein degraders not only exhibit specificity on target degradation and inhibition, also demonstrate their merit to investigate the degradation efficiency dependencies on nucleotide-bound forms and mutation types.

The targeted degradations pave a new avenue to eliminate the oncogenic mutations instead of direct inhibition. As a prerequisite for targeting, the degrader relies on high-affinity ligands that have been developed for binding KRAS. Besides, when applying PROTAC degraders containing a protein ligand, effective delivery into targeted tumor cells will be a rate-limiting step for their future applications in clinics.

#### New directions

Membrane association is important for activation of Ras proteins and most small GTPases. In the case of Ras proteins, the dimerization of Ras at the membrane surface is recognized as a regulating event to induce dimerization of Raf kinases for their activation.^322,323^ Therefore, targeting membrane-association surface of Ras might provide another opportunity to inhibit downstream signaling. Protein ligands, DARPin-K13/K19 and NS1 monobody, bind to the allosteric lobe and demonstrate the inhibitory effects on downstream signaling. NMR characterization of a chemical compound (cmpd2) reveals that the ligand binds to SI/II-P adjacent to Ras-membrane interface and shifts RAS spatial orientation equilibrium, presenting a unique manner to inhibit Ras-Raf signaling.^324^ In another study, compounds generated by the repurposing of fendiline block proliferation of KRAS-driven tumor cells through perturbing membrane localization of KRAS, yet the binding sites of these compounds were not able to be characterized.^325^

Exploring the dynamic properties of switch regions is a promising direction to be pursued. An ensemble-based virtual screen has been developed by using conformational ensembles of switch regions and conserved G-boxes derived from experimental restrains of NMR, which is helpful to find the lowly populated pockets suitable for ligand binding.^49,326^ In a recent work, a strategy using conformation-locking antibodies for molecular probes (CLAMPs) has been developed to stabilize rare conformation by antibodies, which provides an experimental approach for ligand screen and interaction studies based on these rare conformations.^327^

Similar to PROTAC, molecular glues represent a concept able to induce neo-protein-protein associations by small molecule ligands.^328^ Bifunctional ligands were designed by chemically linking two ligands together: one ligand contains a KRAS binding module while the second contains a cyclophilin A/ FKBP12 binding module.^329^ The bifunctional ligand successfully induced the non-native associations of KRAS to cyclophilin A/FKBP12 in cells and blocked KRAS-BRAF interactions in vitro.

At the genetic level, G-quadruplex (G4) sequences in the promoter region of KRAS have been shown to regulate transcription of KRAS. G4 structures are sensitive to changes of microenvironments in the nucleus of tumor cells. The unique structural configurations of G4 likely present a different opportunity to target KRAS.^330–332^ In a recent study, the structure of KRAS G4 in complex with the natural products berberine and coptisine has been determined by NMR, which delineate the structural basis for ligand binding specificity and downregulation of KRAS transcription caused by the ligands.^333^ The complex structures further prove the potential of G4 as a therapeutic target. However, targeting G4 does not render selectivity toward different KRAS oncogenic mutations. Further pharmacological evaluations are required, with G4 possibly acting as a cooperative target for lowering transcription of the oncogenic proteins.

#### Progress of targeting other major small GTPases: Rho, Arf, and Rab

Drug discovery efforts targeting other families of small GTPases are not as advanced as those targeting the Ras family and nearly none have advanced to clinical trials.

Rho proteins, Rac proteins, and CDC42 hold immense interest for developing molecular modulators (activators/inhibitors).^334–336^ Altered expression, rather than aberrant mutations, of Rho family proteins is a major driver of diseases. As such, Rho/GEF interactions are the primary mechanism being targeted. NSC23766 is an early example discovered from virtual screening; it inhibits Rac-GEF interactions through binding to a groove located in Rac/GEF interface and does not interfere with interactions of Rho and CDC42 with their specific GEFs.^337^ NSC23766 inhibits proliferation, anchorage-independent growth, and invasion of the prostate cancer PC-3 cells. EHT 1864, another Rac1 inhibitor, was reported to selectively bind to Rac1 and interfere with guanine nucleotide association. EHT 1864 potently inhibits Rac1 downstream signaling and blocks Rac1-driven transformation of NIH 3T3 cells.^338,339^ ZCL278 is another ligand obtained from virtual screening and specifically binds to CDC42/intersectin (CDC42 GEF) interface. It blocks microspike formation in 3T3 fibroblast cultures, neuronal branching, and actin-based motility and migration in PC-3 cells.^340^ Another ZCL compound, ZCL367, binds to CDC42 with improved selectivity and inhibits proliferation and migration of multiple tumor cell lines, including the lung cancer cells containing EGFR or KRAS G12S mutations.^341^ CASIN (CDC42 activity-specific inhibitor) is another ligand that interferes with GEF-mediated activation. It interrupts CDC42-mediated actin dynamics and induces mobilization of hematopoietic stem cells from bone marrow to peripheral blood in mouse models.^342^ ARN22089 compound is obtained through in-silico screening based on the CDC42/PAK6 complex structure and it recognizes the GTP-bound “on” state of CDC42, blocking the interaction between CDC42 and its downstream effectors.^343^ ARN22089 displays various inhibitory effects on tumor cell growth and angiogenesis in vitro and triggers pro-inflammatory and apoptotic signaling as well as growth of BRAF mutant mouse melanomas through inhibition of MAPK pathway. A series of compounds screened by time-resolved fluorescence methods can block proliferation and migration of breast carcinoma cells through interrupting interactions between CDC42 and the scaffold protein IQGAP1.^344^ In a recent study, a covalent ligand, DC-Rhoin04, has been discovered to bind to an allosteric pocket mainly composed by switch II next to residue C107 in RHOA and inhibits migration and invasion of tumor cells. This binding has been verified and characterized by crystallization.^345^ ML141 binds to CDC42 in a reversible non-competitive manner and inhibits CDC42 selectively. It is reported to suppress division and induce death of basal-like breast cancer cells and enhance their sensitivity to tamoxifen treatment in vivo.^346^

Arf family and their regulatory proteins are important therapeutic targets in cancer treatment.^252^ There are several natural products that have been discovered that bind to and inhibit Arf proteins. Brefeldin A (BFA) is a fungi metabolite identified to significantly alter protein transportations between Golgi and the ER.^347,348^ The structure of ternary complex formed by ARF1, Sec7 catalytic domain of GEF, and BFA corroborates that BFA binds to a hydrophobic pocket at ARF1/Sec7 interface and blocks GTP exchange for activation.^349^ BFA has been previously used as a molecular probe to interrogate functionality of ARF1, but a BFA derivative was recently reported to putatively bind to the ARF1/SEC interface and show potential anti-leukemia activity.^350^ LM11 is an inhibitor that is screened based on the complex structure of ARF1 with Sec7 domain of ARNO (Arf GEF). It binds to the ARF1/Sec7 interface similarly to BFA, inhibits activation of ARF1 rather than ARF6, and blocks both ARF1-mediated trafficking at Golgi and ARNO-dependent migration of Madin-Darby canine kidney cells.^351^ Chlortetracycline (CTC) is a tetracycline-class antibiotic obtained from a high throughput assay using active ARF6 attached on the membrane surface. It is a non-nucleotide-competitive ligand that likely inhibits GEF-mediated activation by binding to an allosteric pocket and blocks ARF6-mediated migration and invasion of breast tumor cell lines.^352^ Of note, membrane association is critical for Arf activation and thus important in evaluating drug actions.^353^

Dysregulations of Rab proteins are involved in many human pathologies, including neurodegenerative disease and bacterial infections. Although Rabs are established as an important category of therapeutic targets, their drug development progress is lagging compared to the Ras and Rho families. More complex regulatory mechanisms, structural and sequence similarities shared between Rab members and other small GTPases families, and the requirements for passing through the blood-brain barrier pose challenges to Rab inhibitor drug discovery.^354^ CID1067700 is a compound obtained from a high throughput screen and acts as a GTP-competitive inhibitor with selectivity towards RAB7 in biochemical assays;^355^ it displays inhibitory effects in disease models of ischemic stroke^356^ and lupus.^356^ NAB2 (N-arylbenzimidazole) was identified to alleviate α-Synuclein induced toxicity in PD cell models, and a following chemoproteomic study reveals that RAB1 is a target of NAB2 for its rescuing effects.^357^ Exploring the allosteric pockets on the Rab protein surfaces has also been pursued based on structure-based computational analysis.^358,359^ Relative to other families, drug discovery efforts toward Ran are limited, though there have been some early attempts to interfere with RAN-GEF interactions.^360,361^ Nevertheless, the altered expression of Ran is closely related to tumorigenesis in different cancer types, indicating the potential of Ran to be the therapeutic target for these tumors.^362^

Taken together, there are many compounds identified as inhibitors or modulators to Rho, Arf, and Rab. Although some of these compounds have been well characterized, most of the molecules were identified through virtual screening or in vitro high throughput assays before being tested with functional assays. Structural characterizations, such as protein-ligand complex structures determined by crystallization or NMR, have been recognized as an indispensable criterion for evaluating a new drug candidate,^363^ but the complex structures for inhibitors binding to Rho, Arf, or Rab are still missing. Considering the experience of targeting KRAS, these molecules can serve as the starting point for further in-depth characterizations and optimizations of small GTPases inhibitors. Future drug development would be accelerated by adopting the versatile strategies developed for targeting KRAS and characterizing the drug actions.

### Indirect targeting

**Fig. 7:**
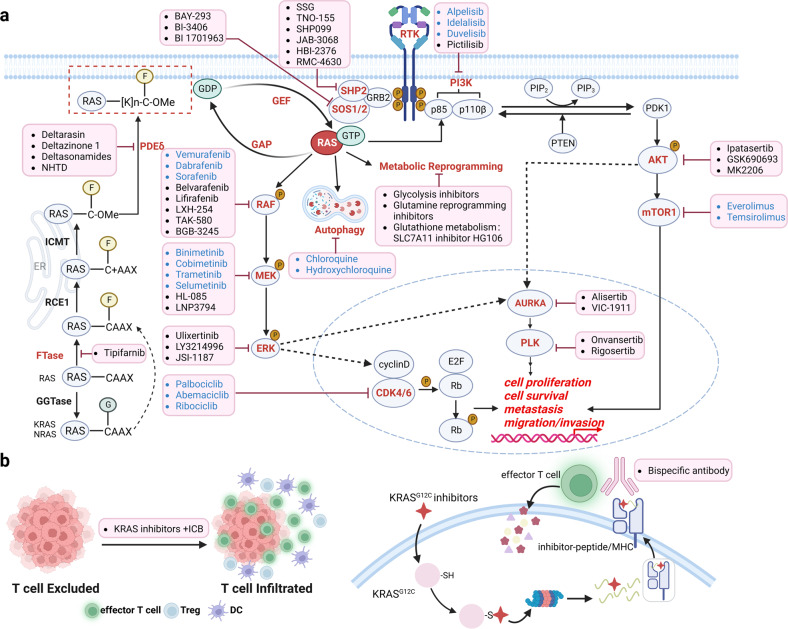
Strategies for indirectly targeting RAS and emerging approaches for targeting oncogenic RAS with immunotherapy. **a** Targeting key post-transcriptional modifications of RAS protein as well as upstream and downstream factors of RAS signaling have been proved as promising strategies for interfering with RAS activity and exhibiting anti-tumor effects in RAS mutant-driven tumors. Inhibitors targeting the key factors controlling RAS post-translational modifications and membrane trafficking (FTase, PDE6δ), upstream mediators (RTK receptors, SHP2, SOS1/2), and downstream effectors (RAF, MEK, ERK, CDK4/6, PI3K, AKT, mTOR1, AURKA, PLK) are listed. FDA-approved drugs are highlighted in blue, others are still under preclinical or clinical study. **b** Combination of KRAS^G12C^ inhibitors with immune checkpoint blockade and development of bispecific antibodies with T cell engager targeting neoepitopes derived from KRAS^G12C^ inhibitor-mediated covalent modification of KRAS^G12C^. This figure was created with BioRender.com

The early efforts to inhibit Ras proteins mainly involve targeting the regulatory mechanisms rather than direct targeting. Presently, indirect targeting represents another important branch to block the abnormal cell proliferation driven by the non-targetable hotspot mutations or serves as a combinatory approach together with direct targeting to enhance the inhibition of Ras downstream pathways and overcome the drug resistance of current KRAS inhibitors. Indirect targeting includes targeting post-translation modifications, membrane association, upstream regulators, downstream effectors, synthetic lethal partners, and metabolic rewiring [Fig. 7a].

#### Targeting the post-translational modifications and membrane trafficking of small GTPases: FTase, PDEδ

Membrane targeting is important for the activation and function of most small GTPases. RAS undergoes a series of post-translational modifications (PTMs) to attach on cell membranes and to subsequently interact with downstream effectors and transduce signals. Therefore, interfering with key PTMs of RAS has been identified as one of the weaknesses of RAS, widening the chemical space in the discovery of RAS inhibitors. Since FTase is the first rate-limiting enzyme of the CAAX processing, it was hypothesized that inhibiting FTase would disrupt RAS cellular location and dampen downstream signaling. Thus, many efforts were made to block the activity of FTase. Unfortunately, although several FTase inhibitors (FTIs) showed great anti-tumor potency in preclinical studies, phase II and phase III clinical trials on solid tumors showed resistance to FTIs.^364–366^ One explanation provided for this disappointing failure is that *KRAS* and *NRAS* mutations are susceptible to alternative prenylation mediated by geranylgeranyltransferase-I (GGTase-I) which promotes RAS membrane localization and oncogenic activation when FTase is blocked.^365,367,368^

Recently, there has been a renewed hope in the use of FTIs in *HRAS*-mutant cancers. HRAS is prenylated exclusively by FTase and it is theoretically much more sensitive to FTIs.^367^ Indeed, a phase II clinical trial (NCT02383927) of FTI tipifarnib showed inspiring anti-tumor activity in patients bearing recurrent and/or metastatic (R/M) head and neck squamous cell carcinoma (HNSCC) with *HRAS* mutations.^369^ This study also found that patients with high mutant *HRAS* variant allele frequency (VAF) have more clinical benefit after tipifarnib administration, which not only supports the breakthrough therapy designation approval of tipifarnib in HNSCC patients with VAF ≥ 20%, but also accelerates the repurposing of tipifarnib in tumor types harboring *HRAS* mutations and demonstrates that VAF of mutant *HRAS* might be a promising biomarker for tipifarnib efficacy.

After post-translational modifications, phosphodiesterase6δ (PDE6δ) binds and escorts farnesylated Ras to the plasma membrane and thereby enhances RAS signaling activity.^370^ This finding calls for the development of inhibitors disrupting the interaction between RAS and the farnesyl-binding pocket of PDE6δ. Deltarasin is the first proven benzimidazole inhibitor targeting the farnesyl-binding site of PDE6δ with *K*_D_ = 7.6 ± 1.3 nM.^371^ In KRAS-driven human pancreatic ductal adenocarcinoma cells (hPDACs), Deltarasin induces decreased membrane localization of KRAS, inhibits downstream MAPK signaling transduction, and significantly inhibits tumor cell growth both in vitro and in vivo. However, Deltarasin was found to show a “switch-like” inhibition of proliferation, which could be explained by its general cytotoxicity or its off-target effect of binding different membrane proteins, such as GPCRs.^372,373^ Deltazinone 1 is another pyrazolopyridazinone inhibitor that has a similar binding method to PDE6δ as Deltarasin.^372^ Deltazinone 1 (*K*_D_ = 8 ± 2 nM) exhibits higher specificity to PDE6δ and less general cytotoxicity than Deltarasin, however, it requires >20 μM to show inhibitory effects on RAS signaling and cell proliferation in cell-based assays. This limitation might be due to the fast release of inhibitors from PDE6δ by the allosteric engagement of release factor Arl2, a small GTPase belonging to ARF family. This suggests that inhibitors with slow off-rates or covalent binding modes could overcome this limitation.^374^ Deltasonamides were later developed as more potent PDE6δ inhibitors with much stronger non-covalent interactions, dramatically reducing release caused by Arl2.^374^ (E)-N′-((3-(tert-butyl)-2-hydroxy-6,7,8,9-tetrahydrodibenzo [b, dfuran-1-yl] methylene)-2,4-dihydroxybenzohydrazide (NHTD) was recently discovered as a new PDE6δ inhibitor via high throughput molecular docking-based screening. NHTD treatment causes KRAS delocalization and showed significant anti-tumor activity in KRAS-driven non-small cell lung cancer (NSCLC) xenografts with good tolerance.^375^

In vitro study identified benzyl indazole compound CHS-111 as a RHOA inhibitor which acts through reducing RHOA membrane association and inhibiting its interaction with GEF Vav.^376^ In addition, through phenotypic screening, Secramine A was reported to repress CDC42 activation and downstream signaling pathway via stabilizing its interaction with RhoGDI1, which prevents CDC42 membrane attachment and GDP-GTP exchange.^377^ Ras proteins are prenylated by FTase or GGTase-I as indicated previously. Rho family and Rab family are generally geranylgeranylated by GGTase-I and II respectively, which facilitates their membrane association and activation. Nitrogen-containing BPs (N-BPs), used to treat skeletal diseases, were found to have inhibitory effect on GGTase-I.^378,379^ Later, N-BP derivatives such as risedronate or zoledronic acid, were shown to induce apoptosis of myeloma or mesothelioma tumor cells in vitro through inhibiting prenylation of Rab proteins.^380,381^

#### Targeting RAS activation by upstream regulators: GEFs, SHP2, STK19

Since mutationally activated RAS was first discovered in human cancer in 1982,^382^ mutations of RAS have been well established as the drivers of diverse types of lethal cancers, such as colorectal cancer, pancreatic cancer, and lung cancer.^383–388^ Oncogenic mutations of RAS usually disrupt the GAP-mediated GTP hydrolysis and lead to constitutive activation. Thus, developing medications to interfere with the upstream events that are responsible to regulate GTPase activities of RAS constitutes an alternative approach to dampen RAS-driven tumor growth.

#### GEFs

Targeting GEFs or other related regulatory protein kinases that regulate RAS activity is an alternative anti-RAS strategy. Particularly, son of sevenless 1 (SOS1) has become a target that gained focus in RAS-driven tumor therapy. SOS1 is a major GEF for RAS^389^ and essential for RAS activation by facilitating GDP-GTP exchange. Thus, interfering with SOS1-mediated RAS activation is a strategy to inhibit RAS. The first SOS1 modulator was identified by serendipity and subsequent cocrystal analysis. It was shown to bind to a hydrophobic pocket in the CDC25 domain adjacent to the catalytic site of SOS1, which interacts with Switch II region of RAS.^390^ The early generation SOS1 modulators are interestingly activating RAS-mediated MAPK pathways instead of inhibiting it in cells,^390–395^ thus the development of RAS-inhibitors by targeting SOS1 has been rudimentary. Until recently, the discovery of quinazoline molecules, including BAY-293 from Bayer^396^ and BI-3406 from Boehringer Ingelheim^397^ shed light on the development of SOS1 inhibitors. Intriguingly, these two inhibitors both targeted the same hydrophobic pocket that previously reported to cause SOS1 mediated RAS activation. The mechanism of action (MOA) for their inhibition of SOS1 are identical, which could be explained by disrupting the RAS binding epitope on SOS1 interaction sterically.^396,398^ BAY-293 was reported to show anti-proliferative activity in both *KRAS* mutant and wild-type human cancer cell lines, and the efficacy of this could be enhanced by the combination of ARS-853, a covalent inhibitor of KRAS^G12C 396^. BI-3406 dampens the key interaction between Tyr884^SOS1^-Arg73^RAS^ for RAS activation, exhibits improved potency in KRAS-driven tumors both in vitro and in vivo, and synergizes with MEK inhibitor trametinib, resulting in remarkable tumor regressions with adequate tolerance.^397,398^ BI 1701963, the analog of BI-3406, is the only SOS1 inhibitor that has moved forward into clinical study. BI 1701963 alone or in combination with trametinib (NCT04111458), KRAS^G12C^ inhibitor Adagrasib (NCT04975256), and with KRAS^G12C^ inhibitor BI 1823911 as well as midazolam (NCT04973163) are now in phase I clinical studies. However, BI 1701963 alone or in combination with irinotecan in patients with advanced bowel cancer with *KRAS* mutations (NCT04627142) and BI 1701963 alone or in combination with MEK inhibitor BI 3011441 in patients with *KRAS* mutant non-small cell lung cancer and colorectal cancer (NCT04835714) were terminated recently due to the unexpected toxicity. Combinatorial treatment strategy is well characterized to prevent or delay resistance and improve antitumor efficacy. However, the termination of the indicated clinical trials warns us that recognizing and minimizing combination-induced toxicity is one of the main challenges for developing combinatory therapy in clinic.^399,400^ Designing trials that reflect toxicity and intermittent dosing in early clinical trials^400^ may provide great insight to strike a balance between efficacy and toxicity.

Considering advancements made on targeting Ras proteins, other small GTPases have attracted increased attention since new approaches to target small GTPases have been developed. Some of these methods include disrupting the interaction between small GTPases and GEFs, inhibiting nucleotide binding, impairing GEF-regulated guanine nucleotide exchange, and interfering with membrane binding. Most small molecule inhibitors in this field are based on the strategy that inhibits the binding of small GTPases to their GEFs. For example, Y16 was identified to specifically inhibit RHOA activation through disrupting Rho GEF LARG binding to RhoA.^401^ LM11, a noncompetitive ARF1 inhibitor, targets the binding of ARF1 to GEF ARNO at the Golgi and significantly decreased metastatic capability of breast cancer cells in a zebrafish model.^351,402^ Similar to quinazoline inhibitors binding to SOS1, Sec7 inhibitor H3 (SecinH3) can also disrupt the interaction of ARF1 and ARF6 with ARNO by inhibiting the Sec7 catalytic domain in ARNO. This was later found to show benefits in treating breast tumors,^403^ colorectal tumors^404^ and non-small-cell lung tumors^405^ both in vitro and in vivo. NSC23766 was characterized as a small molecule that binds to a putative binding pocket in Rac1 that interacts with Rac-GEFs Trio and Tiam-1 rather than Vav,^337^ and it can significantly impede BCR-ABL-induced myeloproliferative disease in xenograft model in vivo.^406^ Further studies showed that NSC23766 derivative EHop-016 potently inhibits Rac at low micromolar levels (IC_50_ = 1.1 μM) via binding to a deeper pocket in Rac and disrupting the key interactions between Rac1 and Rac-GEF Vav1.^407^ This inhibitor holds promise for treating metastatic cancers evidenced by its effective anti-tumor activity in multiple in vitro and in vivo studies.^407–410^ AZA1, which is also a derivative of NSC23766, inhibits the binding of Cdc42 and Rac1 with their GEFs.^411^ In a preclinical advanced colon cancer mouse model, AZA197, an AZA1 analog with improved selectivity on Cdc42, delayed tumor growth and significantly prolonged mouse survival via inhibiting Cdc42 and its downstream PAK1 signaling pathway.^412^

#### GAPs

Stimulating RAS hydrolysis through activating RAS-specific GAPs, such as p120GAP, neurofibromin (NF1), and RGS3, is an essential regulation to turn off the activated RAS.^51,413–415^ Most RAS mutant alleles show reduced intrinsic GTPase activity in comparison with WT RAS, except for *KRAS*^*G12C*^ allele.^416^ Moreover, RAS mutation impairs GAP-mediated GTP hydrolysis, leading to constitutive RAS activation. RGS3, a conventional GAP for heterotrimeric G protein α subunit (Gα), has been recently discovered with capacity in catalyzing GTP-hydrolysis for WT and mutated KRAS, including G12C, G12V, G12D, G13D, and G13V.^51,417^ Compared with canonical Ras-GAP, RGS3 catalyzes GTP hydrolysis of KRAS through a conserved asparagine residue instead of the arginine finger. Current G12C inhibitors recognize and interact exclusively with the GDP-bound protein, but the majority of the G12C mutant remains binding to GTP in tumor cells due to defective GAP mediated hydrolysis. Thus, RGS3 has been established as an alternative GAP to return the G12C mutant back to the GDP-bound “OFF” state for inhibitor actions. Despite a slower hydrolysis rate mediated by RGS3 compared to RAS-GAP, discovery of a regulatory role of a large G-protein GAP in GTP hydrolysis of KRAS presents a new angle to understand biochemical properties of the oncogenic mutants and might create a new opportunity for alleviating KRAS-driven tumorigenesis.

#### SHP2

Src homology-2-containing protein tyrosine phosphatase 2 (SHP2) serves as a vital regulatory node that activates RAS/MEK/ERK signaling and promotes proliferation of tumor cells by either recruiting GRB2-SOS1 complex to plasma membrane or directly dephosphorylating RAS at Y32.^418^ Of note, recent studies demonstrated the essential role of SHP2 in KRAS-driven tumor progression, showing that genetic ablation or pharmacological inhibition of SHP2 did dramatically delay tumor growth in *KRAS* amplified gastric carcinoma, Kras^G12D^ driven pancreatic tumor, and NSCLC tumor models with *Kras*^*G12D*^ or *Kras*^*G12V*^ mutation, This demonstrates that targeting upstream SHP2 is a potential approach to inhibit RAS activation, including wild-type RAS amplification and RAS mutants.^419–422^ Furthermore, these three studies also noted that SHP2 is tightly associated with the resistance caused by MEK inhibitors and its inhibition in conjunction with MEK inhibitor treatment blocks the rebound of MAPK signaling and thus results in a more potent anti-tumor effect. Presently, there are fifteen SHP2 inhibitors that have moved into clinical trials and most of them are still in the early stages. According to the binding mode, SHP2 inhibitors can be classified into two categories: orthosteric inhibitors and allosteric inhibitors. Sodium stibogluconate (SSG) is the first orthosteric SHP2 inhibitor. Given that SSG can synergize with interferon-α2b (IFN-α2b), phase I clinical trials studying the combination of SSG and IFN-α2b in the treatment of advanced tumors (NCT00629200) and stage IV melanoma (NCT00498979) were launched and completed in 2018 and 2020, respectively. However, no objective regression of disease was observed in the trials^423^ and no follow-up research has been reported since then. TNO-155, a derivative of SHP099, is the first allosteric SHP2 inhibitor that entered the clinical trial stage. There have been eight clinical trials for TNO-155 mono- or combinational treatment for different types of tumors. A phase I study was launched in April 2017 that aimed to find the recommended dose for TNO-155 in combination with EGFR inhibitor nazartinib in patients with advanced solid tumors, including *EGFR* mutant NSCLC and *KRAS*^*G12*^-mutant NSCLC (NCT03114319). Efficacy of TNO-155 in combination with PD-1 antibody spartalizumab or CDK4/6 inhibitor ribociclib in various types of advanced tumors is under evaluation in a phase I study (NCT04000529). In addition, a phase I study of TNO-155 in combination with KRAS^G12C^ inhibitor Adagrasib in patients suffering from advanced solid tumors that harbor *KRAS*^*G12C*^ mutations was initiated in April 2020 (NCT04330664). Additionally, a phase Ib/II study of TNO-155 with another KRAS^G12C^ inhibitor JDQ443 in *KRAS*^*G12C*^ mutant solid tumors is currently recruiting participants (NCT04699188). Concurrently, other SHP2 allosteric inhibitors, such as JAB-3068, HBI-2376, RMC-4630 etc. are in phase I trials. Of note, a phase I study on HBI -2376 in patients with advanced solid tumors harboring *KRAS* or *EGFR* mutations are now recruiting (NCT05163028) (Table 2) and RMC-4630 combined with LY3214996 (NCT04916236) and Sotorasib (NCT05054725) for the treatment of tumors harboring *KRAS* mutations are under phase I and phase II trials, respectively (Table 3).

**Table 2:** Agents targeting RAS mutant-tumors

Drug	Biomarker	Disease Setting^a^	ClinicalTrials.gov
KRAS^G12C^ inhibitors			
Sotorasib	*KRAS*^*G12C*^ Mutation	NSCLC	approved for previously treated KRAS G12C mutated NSCLC, ongoing trials: NCT04625647, NCT05398094, NCT05311709
Sotorasib	*KRAS*^*G12C*^ Mutation	Advanced or Metastatic Solid Tumors	NCT04380753
Adagrasib	*KRAS*^*G12C*^ Mutation	NSCLC	approved for previously treated KRAS G12C mutated NSCLC, ongoing trials: NCT04685135,NCT04613596
Adagrasib	*KRAS*^*G12C*^ Mutation	Advanced Solid Tumors	NCT03785249, NCT05263986
LY3537982	*KRAS*^*G12C*^ Mutation	Solid Tumors	NCT04956640
JDQ443	*KRAS*^*G12C*^ Mutation	NSCLC	NCT05132075, NCT05445843
JDQ443	*KRAS*^*G12C*^ Mutation	Advanced Solid Tumors	NCT04699188
JAB-21822	*KRAS*^*G12C*^ Mutation	NSCLC	NCT05276726
JAB-21822	*KRAS*^*G12C*^ Mutation	Advanced Solid Tumors	NCT05009329, NCT05002270
D-1553	*KRAS*^*G12C*^ Mutation	NSCLC	NCT05383898
D-1553	*KRAS*^*G12C*^ Mutation	Advanced Solid Tumors	NCT04585035
YL-15293	*KRAS*^*G12C*^ Mutation	Advanced Solid Tumor	NCT05119933, NCT05173805
GFH925	*KRAS*^*G12C*^ Mutation	Advanced Solid Tumors	NCT05005234
GDC-6036	*KRAS*^*G12C*^ Mutation	NSCLC	NCT03178552
GDC-6036	*KRAS*^*G12C*^ Mutation	Advanced Solid Tumors	NCT04449874
LY3537982	*KRAS*^*G12C*^ Mutation	Advanced Solid Tumors	NCT04956640
BI 1823911	*KRAS*^*G12C*^ Mutation	Advanced Solid Tumors	NCT04973163
MK-1084	*KRAS*^*G12C*^ Mutation	Advanced Solid Tumors	NCT05067283
GH35	*KRAS* Mutation	Advanced Solid Tumors	NCT05010694
MEK inhibitors			
Binimetinib	*BRAF*^*V600*^ or *NRAS* Mutations	Metastatic Melanoma	NCT01320085
LNP3794	*NRAS* or *KRAS* Mutation	Advanced or Metastatic Solid Tumors	NCT05187858
Cobimetinib	RAS Pathway Mutations	CMML	NCT04409639
HL-085	*NRAS* Mutation	Melanoma	NCT05217303
Cancer vaccines			
RNA Tumor vaccine	*KRAS* Mutation	Advanced Solid Tumors	NCT05202561
mDC3/8-KRAS Vaccine	*KRAS* Mutation	PDAC	NCT03592888
Pooled Mutant KRAS-Targeted Long Peptide Vaccine	*KRAS* Mutation	NSCLC	NCT05254184
ELI-002 2 P	*KRAS* Mutation	Solid Tumors	NCT04853017
SHP2 inhibitors			
TNO155	*EGFR* or *KRAS*^*G12C*^ Mutation	Advanced Solid Tumors	NCT03114319
HBI-2376	*KRAS* or *EGFR* Mutations	Solid Tumors	NCT05163028
RMC-4630	*KRAS* amplifications, *KRAS*^*G12C*^ (NSCLC), *BRAF* Class 3, or *NF1*^*LOF*^ (NSCLC and gynecological cancers) mutations	Relapsed or Refractory Solid Tumors	NCT03634982
GDC-1971	*KRAS*, *BRAF* or *MEK* Mutation	Advanced or Metastatic Solid Tumors	NCT04252339
SOS1 inhibitor			
BI 1701963	*KRAS* Mutation	Solid Tumors	NCT04111458
EGFR antibody			
Cetuximab	*APC*, *TP53* and *RAS* Mutation	CRC	NCT04853043
FTase inhibitors			
Tipifarnib	*HRAS* Mutation	NSCLC	NCT03496766
Tipifarnib	*HRAS* Mutation	Thyroid cancer, HNSCC	NCT02383927
Tipifarnib	*HRAS* Mutation	Advanced Solid Tumors, Lymphoma, or Histiocytic Disorders	NCT04284774
Adoptive cell therapies			
Anti-RAS-G12D mTCR	HLA-A11:01 *RAS*^*G12D*^ Mutation	Advanced solid Tumors	NCT03745326
Anti-RAS-G12V mTCR	HLA-A11:01 *RAS*^*G12V*^ Mutation	Advanced solid Tumors	NCT03190941
RAF inhibitors			
KIN-2787	*BRAF* and/or *NRAS* Mutation	Solid Tumors	NCT04913285
BGB-283	*BRAF, KRAS* or *NRAS* Mutation	Advanced or Metastatic Solid Tumors	NCT02610361
BGB-3245	*BRAF, KRAS* or *NRAS* Mutation	Advanced or Metastatic Solid Tumors	NCT04249843
TAK-580	RAS Pathway Mutations	Solid Tumors	NCT03429803
RNA interfering drug			
NBF-006	*KRAS* Mutation	NSCLC, PDAC, CRC	NCT03819387
DNA synthesis inhibitors			
Capecitabine	*KRAS* Mutation	PMP	NCT05321329
CDK4/6 inhibitors			
Abemaciclib	*KRAS* Mutation	NSCLC	NCT02152631
FASN inhibitors			
TVB-2640	*KRAS* Mutation	NSCLC	NCT03808558

^a^*CRC* colorectal cancer, *CMML* chronic myelomonocytic leukemia, *HNSCC* head and neck squamous cell carcinoma, *NSCLC* non-small-cell lung cancer, *PDAC* pancreatic ductal adenocarcinoma, *PMP* pseudomyxoma peritonei

**Table 3:** Combination strategies targeting RAS-mutant tumors

Combination Targets	Drug	Biomarker	Disease Setting^a^	Clinical Trials.gov
KRAS^G12C^ inhibitor Combinations				
PD-1/PD-L1 antibody	Sotorasib and PD-1 /PD-L1 antibodies	*KRAS*^*G12C*^ Mutation	Advanced NSCLC	NCT03600883
	Adagrasib and Pembrolizumab	*KRAS*^*G12C*^ Mutation	Advanced or Metastatic NSCLC	NCT04613596
	GDC-6036 and Atezolizumab	*KRAS*^*G12C*^ Mutation	Advanced Solid Tumors	NCT04449874
SOS1 inhibitor	Adagrasib and BI 1701963	*KRAS*^*G12C*^ Mutation	NSCLC, CRC	NCT04975256
	BI 1823911 and BI 1701963	*KRAS* Mutation	Solid Tumors	NCT04973163
Chemotherapy	Sotorasib and Cisplatin/Carboplatin and Pemetrexed	*KRAS*^*G12C*^ Mutation	Lung Cancer	NCT05118854
	Sotorasib and Docetaxel	*KRAS*^*G12C*^ Mutation	NSCLC	NCT04303780
	Sotorasib and Liposomal Irinotecan and 5 Fluorouracil + Leucovorin	*KRAS*^*G12C*^ Mutation	PDAC	NCT05251038
	Sotorasib and Gemcitabine + Nab-paclitaxel	*KRAS*^*G12C*^ Mutation	PDAC	NCT05251038
SHP2 inhibitor	Sotorasib and RMC-4630	*KRAS*^*G12C*^ Mutation	NSCLC	NCT05054725
	JAB-21822 and JAB-3312	*KRAS*^*G12C*^ Mutation	Advanced Solid Tumors	NCT05288205
	Adagrasib and TNO155	*KRAS*^*G12C*^ Mutation	Advanced or Metastatic solid Tumors	NCT04330664
	JDQ443 and TNO155	*KRAS*^*G12C*^ Mutation	Advanced solid Tumors	NCT04699188
	GDC-6036 and GDC-1971	*KRAS*^*G12C*^ Mutation	Advanced Solid Tumors	NCT04449874
EGFR inhibitor	GDC-6036 and Cetuximab	*KRAS*^*G12C*^ Mutation	Advanced Solid Tumors	NCT04449874
	GDC-6036 and Erlotinib	*KRAS*^*G12C*^ Mutation	Advanced Solid Tumors	NCT04449874
	JAB-21822 and Cetuximab	*KRAS*^*G12C*^ Mutation	Advanced CRC	NCT05194995
	Sotorasib and Panitumumab	*KRAS*^*G12C*^ Mutation	CRC	NCT05198934
RAF/MEK Inhibitor	Sotorasib and VS-6766	*KRAS*^*G12C*^ Mutation	NSCLC	NCT05074810
HER inhibitor	Sotorasib and Tarloxotinib	*KRAS*^*G12C*^ Mutation	NSCLC	NCT05313009
CDK 4/6 inhibitor	Adagrasib and Palbociclib	*KRAS*^*G12C*^ Mutation	Advanced Solid Tumor	NCT05178888
ERK inhibitor Combinations				
CDK 4/6 inhibitor	LY3214996 and Abemaciclib	*BRAF* or *RAS* Mutations	Advanced or Metastatic solid Tumors	NCT02857270
Chemotherapy	LY3214996 and Nab-Paclitaxel and Gemcitabine	*BRAF* or *RAS* Mutations	Advanced or Metastatic solid Tumors	NCT02857270
RAF and EGFR inhibitors	LY3214996 and Encorafenib + Cetuximab	*BRAF* or *RAS* Mutations	Advanced or Metastatic solid Tumors	NCT02857270
Autophagy	Ulixertinib and Hydroxychloroquine	*RAS*, non- *BRAF*^*V600*^, *ERK*, or *MEK* Mutations	Advanced Gastrointestinal Malignancies	NCT05221320
RAF inhibitor	JSI-1187 and Dabrafenib	MAPK pathway Mutations	Advanced Solid Tumors	NCT04418167
MEK inhibitor Combinations				
EGFR inhibitor	Binimetinib and Erlotinib	*KRAS* or *EGFR* Mutations	NSCLC	NCT01859026
FGFR inhibitor	Binimetinib and Futibatinib	*KRAS* Mutation	Advanced or Metastatic Solid Tumors	NCT04965818
ALK inhibitor	Trametinib and TPX-0005	*KRAS* Mutation	Advanced or Metastatic Tumors	NCT05071183
Multi-targeting tyrosine kinase inhibitor	Trametinib and Anlotinib	*KRAS* Mutation	NSCLC	NCT04967079
SOS1 inhibitor	Trametinib and BI 1701963	*KRAS* Mutation	Solid Tumors	NCT04111458
Chemotherapy	Selumetinib and Docetaxel	*KRAS* Mutation	Advanced or Metastatic NSCLC	NCT01933932
CDK4/6 inhibitor	PD-0325901 and Palbociclib	*KRAS* Mutation	Solid Tumors	NCT02022982
CDK4/6 inhibitor	Binimetinib and Palbociclib	*KRAS* or *NRAS* Mutation	Metastatic CRC	NCT03981614
Autophagy	Trametinib and Hydroxychloroquine	*KRAS* Mutation	Biliary Cancer	NCT04566133
Autophagy	Binimetinib and Hydroxychloroquine	*KRAS* Mutation	NSCLC	NCT04735068
Autophagy and PD-L1 antibody	Cobimetinib and Hydroxychloroquine + Atezolizumab	*KRAS* Mutation	Gastrointestinal Cancer	NCT04214418
RAF/MEK inhibitor Combinations				
FAK inhibitor	VS-6766 and Defactinib	*KRAS* Mutation	NSCLC	NCT04620330
RTK inhibitor	VS-6766 and Cetuximab	*KRAS* Mutation	Advanced CRC	NCT05200442
RAF inhibitor Combinations				
MEK inhibitor	Lifirafenib and PD-0325901	*KRAS* Mutation	Solid Tumor	NCT03905148
	Belvarafenib and Cobimetinib	*RAS* or *RAF* Mutations	Advanced or Metastatic Tumors	NCT03284502
	lifirafenib and PD-0325901	*KRAS*-mutant NSCLC or endometrial cancer	Advanced or Metastatic Tumors	NCT03905148
	LXH-254 and Trametinib	*KRAS*-mutant or *BRAF*-mutant NSCLC or *NRAS*-mutant melanoma	Advanced or Metastatic Tumors	NCT02974725
	LXH-254 and Trametinib	*BRAF*^*V600*^ or *NRAS* Mutation	Melanoma	NCT04417621
	Dabrafenib and Trametinib	*RAS* or *BRAF*^*V600*^ Mutation	Metastatic DTC	NCT03244956
PD-1 antibody	LXH-254 and PD-1 antibody	*NRAS*-mutant Melanoma and *KRAS*-Mutation	Advanced or Metastatic Tumors	NCT02607813
ERK1/2 inhibitor	LXH-254 and LTT462	*KRAS*-mutant or *BRAF*-mutant NSCLC or *NRAS*-mutant Melanoma	Advanced or Metastatic Tumors	NCT02974725
	LXH-254 and LTT462	*BRAF*^*V600*^ or *NRAS* Mutation	Melanoma	NCT04417621
CDK4/6 inhibitor	LXH-254 and Ribociclib	*BRAF*^*V600*^ or *NRAS* Mutation	Melanoma	NCT04417621
	LXH-254 and Ribociclib	*KRAS*-mutant or *BRAF*-mutant NSCLC or *NRAS*-mutant Melanoma	Advanced or Metastatic Tumors	NCT02974725
SHP2 inhibitor Combinations				
ERK inhibitor	RMC-4630 and LY3214996	*KRAS* Mutation	PDAC, CRC, NSCLC	NCT04916236
PD-1 inhibitor	TNO155 and Spartalizumab	*EGFR or* WT *ALK* NSCLC	Advanced solid Tumors	NCT04000529
CDK4/6 inhibitor	TNO155 and Ribociclib	WT *EGFR* or WT *ALK* NSCLC, *KRAS*-mutant CRC or NSCLC	Advanced solid Tumors	NCT04000529
HER2/EGFR inhibitor Combinations				
mTOR inhibitor	Neratinib and Everolimus	*EGFR* Mutation, *HER2* Mutation, or *HER3/4* Mutation or *KRAS* Mutation	Advanced Malignant Solid Neoplasm	NCT03065387
CDK4/6 inhibitor	Neratinib and Palbociclib	*EGFR* Mutation, *HER2* Mutation, or *HER3/4* Mutation or *KRAS* Mutation	Advanced Malignant Solid Neoplasm	NCT03065387
MEK inhibitor	Neratinib and Trametinib	*EGFR* Mutation, *HER2* Mutation, or *HER3/4* Mutation or *KRAS* Mutation	Advanced Malignant Solid Neoplasm	NCT03065387
PLK inhibitor Combinations				
PD-1 antibody	Rigosertib and Nivolumab	*KRAS* Mutation	NSCLC	NCT04263090
Apoptosis inducer Combinations				
Chemotherapy	Cisplatin and Pemetrexed	*KRAS* Mutation	NSCLC	NCT02743923
anti-PD-1 therapy Combinations				
Chemotherapy	Cyclophosphamide and Fludarabine + Anti-PD-1 monoclonal Antibody	*KRAS*^*G12V*^ Mutation	PDAC	NCT04146298
Cancer vaccine	Pembrolizumab and mRNA-5671	*HLA- A11:01* and/or *HLA- C08:02*; *KRAS*^*G12C*^, *KRAS*^*G12D*^, *KRAS*^*G12V*^ or *KRAS*^*G13D*^ Mutation	NSCLC, non- MSI-H CRC,	NCT03948763

^a^*CRC* colorectal cancer, *DTC* differentiated thyroid cancer, HLA human leukocyte antigen, *MSI-H* microsatellite instability-high, *NSCLC* non-small-cell lung cancer, *PDAC* pancreatic ductal adenocarcinoma

#### STK19

Recently, a serine/threonine kinase, STK19, has been identified as a new NRAS activator by phosphorylating NRAS and subsequently enhancing the downstream NRAS-driven malignant transformation in melanoma cells. Knockdown STK19 by shRNAs has consistently inhibited NRAS activity, downstream signaling, and melanomagenesis in vivo.^424,425^ One of the STK19 inhibitors, ZT-12-037-01, was developed based on an initial hit from a chemical screen. ZT-12-037-01 is competitive inhibitor of ATP for STK19 with IC_50_ = 35 nM. The natural product chelidonine was also found to be a potent STK19 inhibitor with IC_50_ = 125.5 ± 19.3nM.^425,426^ Chelidonine not only suppresses NRAS-mediated signaling, but also significantly inhibits the growth of tumors with NRAS mutation. The discovery of ZT-12-037-01 and chelidonine opens the door of drug discovery utilizing STK19 for inhibition of *NRAS* mutant-driven tumors and provides valuable molecular probes for further investigation of the biological function of STK19. The progress on SHP2 and STK19 also proves that targeting well-characterized PTMs of Ras proteins and other small GTPases is a promising direction for indirect inhibition.

#### Targeting downstream effectors

For RAS, the best-characterized pathways include RAF-MEK-ERK and PI3K-AKT-mTOR, so the downstream effectors in these pathways are important drug targets in cancer treatments as well. Active RAS binds to RAF kinases, stimulating RAF dimerization and subsequent phosphorylation and activation, which leads to activation of MEK-ERK kinase cascade. This is an essential pathway regulating cell proliferation, survival, cell cycle progression, and differentiation. Multiple studies have validated RAF as the key mediator in mutant RAS-driven cancer growth and progression.^427–429^ However, current RAF inhibitors approved for treating *BRAF*^*V600E*^ mutant melanoma are not effective in targeting RAS mutant cancer due to their unique pharmacologic features. They can specifically inhibit BRAF^V600E^ monomers in BRAF mutant cancer cells, but in RAF WT cells, their binding to RAF induces RAF dimerization and allosteric transactivation of the second protomer, leading to paradoxical ERK signaling activation.^430,431^

Next-generation pan-RAF inhibitors, including LY3009120, belvarafenib (clinicaltrials.gov identifier NCT03284502), LXH-254 (NCT02607813, NCT04417621, NCT02974725, NCT03333343, and NCT04294160), lifirafenib (NCT03905148), BGB-3245 (NCT04249843), and TAK-580 (NCT03429803), can also induce WT RAF dimerization. However, they can bind both protomers of RAF dimer with equal potency and inhibit ERK signaling in RAS mutant cells.^432–437^ RAF dimer inhibitors have been reported to inhibit RAS mutant tumors in preclinical models, and several of them have been associated with partial responses in RAS-mutant cancer patients in phase I clinical trials. Two confirmed partial responses out of ten *NRAS*-mutant melanoma patients have been observed following belvarafenib treatment.^432^ Two patients (one endometrial cancer and one NSCLC) out of 66 with *KRAS* mutations had confirmed responses to lifirafenib treatment.^438^ Future large-scale clinical trials are warranted to validate their clinical efficacy. Outside of directly targeting RAF or RAF mutants, rigosertib was recently identified as an effective agent blocking the interaction between RAS-RAS-binding domain (RBD) and RAF family members.^439^ This finding opens a new door for inactivating RAS signaling.

MEK1/2 are the only well-characterized downstream substrates of RAF, making them attractive targets for treating RAS mutant cancer. Current allosteric MEK inhibitors are clinically approved for treatment of *BRAF*^*V600E*^ mutant cancer either alone or in combination with RAF inhibitors.^440^ However, MEK inhibitors have only exhibited modest efficacy against RAS mutant tumors in clinical trials, with *NRAS* mutant melanoma being the only subtype showing responsiveness to MEK inhibition. Twenty percent of *NRAS* mutant-driven melanoma patients had partial response to MEK inhibitor binimetinib in a phase II clinical trial.^441^ Similarly, targeting ERK kinase, the downstream effector of MEK, failed to show clinical benefits in treating RAS mutant cancer. ERK inhibitor ulixertinib resulted in 18% partial response in *NRAS* mutant melanoma patients in a phase I clinical trial. Other ERK inhibitors have not been associated with objective tumor responses in RAS mutant tumors.^442^

There are two reasons that account for the limited efficacy of RAF dimer inhibitors, MEK inhibitors, and ERK inhibitors against RAS mutant tumors: upstream feedback release-induced MAPK pathway reactivation upon RAF dimer inhibitors/MEK inhibitors/ERK inhibitors treatment and limited on-target activity at maximum tolerated doses of the above inhibitors. MAPK pathway is tightly regulated through both transcriptional and post-translational mechanisms. Under normal physiological conditions, ERK activation causes feedback inhibition of RTK, RAS GEF SOS, RAF, and MEK through ERK-mediated phosphorylation. It also activates the transcription of negative regulators of the MAPK pathway including SPRY, SPRED, and DUSPs to ensure a controlled amplitude and duration of ERK signaling. Following treatment with RAF dimer inhibitors, MEK inhibitors, or ERK inhibitors, the MAPK pathway is inhibited, leading to the release of ERK-dependent negative feedback and reactivation of the pathway in the presence of the above inhibitors. On the other hand, studies from genetically engineered mouse models have shown that knock-out of either MEK1/2 or ERK1/2 led to embryonic lethality due to severe intestinal defects, underscoring that MAPK pathway is essential in normal tissue homeostasis. Inhibition of MAPK pathway by RAF dimer inhibitors/MEK inhibitors/ERK inhibitors will cause significant on-target toxicities to normal cells, which limits their efficacy in blocking the pathway in tumor cells.

Since single-agent RAF, MEK, or ERK inhibitors show limited efficacy in treating tumors with RAS mutations, combination strategies including vertical inhibition of multiple members (RAF, MEK, ERK, CDK4/6) in MAPK pathway, autophagy inhibitors hydroxychloroquine, chemotherapy, and immune checkpoint blockade, are being tested in the clinic. The PI3K-AKT-mTOR pathway is also a well-established downstream pathway of RAS. Binding of RAS to PI3K leads to activation of AKT and mTOR, which controls multiple cellular activities through regulation of protein translation and cell metabolism. Mutations in PI3K pathway including PI3K mutations, AKT mutations and amplification, or PTEN loss, are found to be concurrent with RAS mutations in human cancer, indicating that RAS activation may not be sufficient to fully activate PI3K signaling. Activation of PI3K-AKT-mTOR pathway has been reported to contribute to both intrinsic and acquired resistance to MAPK pathway inhibitors, which provides strong rationale for combinatory inhibition of MAPK and PI3K pathway in the treatment of RAS mutant cancer. Presently, alpelisib is the only PI3K inhibitor being utilized for the treatment of breast cancer, while leniolisib was recently assigned as new drug for activated PI3K Delta Syndrome (APDS). However, the safety concerns related to toxicity including hyperglycemia, rash, and diarrhea, caused by on-target effect and off-target effects of PI3K inhibitors are not negligible.^443,444^ Despite synergy observed in combination treatment in preclinical mouse models, clinical trials evaluating the efficacy of combined inhibition of MAPK and PI3K pathway failed due to toxicity in normal tissue. Isoform or mutant allele specific inhibitors with a wide therapeutic window will likely be better tolerated and more effective in targeting MAPK and PI3K signaling in RAS mutant cancer patients.

#### Targeting synthetic lethal partners in RAS mutant cancer

Synthetic lethal screens have been used to identify genes which are indispensable for the survival of RAS mutant cells but not RAS wild-type cells. Early RNAi screens using isogenic cell models or panels of *KRAS* mutant/WT cancer cell lines to identify RAS synthetic lethal genes yielded different targets among different studies. This high variability between independent screens could be attributed to the off-target effects in RNAi libraries. Later CRISPR-CAS9-based screens with lower off-target activities led to identification of regulators which are essential for RAS membrane localization including RCE1 and ICMT, RAS downstream effectors such as CRAF, and regulators of RAS-MAPK pathway such as SHOC2.^428^ Further studies employed in vivo CRISPR-CAS9 screen in either 3D organoid, tumor spheroid, or tumor xenograft models and found synthetic vulnerabilities including TGFBR2, TSC1, regulator of ferroptosis GPX4, and metabolic genes (NADK, KHK) in the context of mutant KRAS activation.^445–447^ However, none of the synthetic lethal targets have been able to transform into successful clinical approaches.

More recently, synthetic lethal screens have been employed to identify targets that function cooperatively with therapies targeting MAPK pathway including MEK inhibitors and KRAS^G12C^ inhibitors in RAS mutant cancer cells. Since the efficacy of MEK or KRAS^G12C^ inhibitors monotherapy in RAS mutant cancer is limited due to feedback reactivation of RAS downstream pathways, identification of potential combination strategies through synthetic lethal screens could be the key to achieve maximum and durable suppression of RAS signaling in tumors and improve patient response. Combinatorial CRISPR-CAS9 screen with MEK inhibitor treatments in RAS mutant cancer cells identified RTK signaling, anti-apoptotic proteins MCL1 and BCL2L1, and Centrosome-associated protein CENPE as synthetic targets which contribute to MEK inhibitor resistance.^448–450^ Interestingly, SHOC2 was identified as synthetic lethality again in *KRAS* mutant-driven lung and pancreatic cancer following MEK inhibitor treatment, which further validated its essential role in the context of RAS activation.^448^ The clinical success of KRAS^G12C^ inhibitors evoked a plethora of enthusiasm in finding synthetic lethal genes whose depletion will enhance sensitivity of KRAS^G12C^ mutant cancer cells to KRAS^G12C^ inhibitors. These efforts uncovered RTK such as IGF1R and EGFR, CPD, and Aurora kinase as potential therapeutic targets in combination with KRAS inhibitors.^451,452^

To date, synthetic lethal studies employing large panels of RAS mutant cell lines combined with 3D culture models or xenograft models to mimic in vivo tumor microenvironment have identified many RAS synthetic lethal targets. Increasing evidence indicates that the synthetic lethal vulnerabilities in RAS mutant tumors are unique to specific contexts of genetic background, cell lineage, different RAS mutant alleles, and tumor microenvironment.^453^ Thus, future synthetic lethal studies should consider the specific tumor context to better understand RAS dependencies during tumor initiation, progression, and drug resistance development.

#### Targeting autophagy

Research over the past decade has identified that RAS activation has a critical role in reprogramming metabolic process that promotes tumorigenesis and survival of tumor cells. Therefore, targeting metabolic alterations induced by mutant RAS is of great significance. In 2011, scientists found that introduction of *HRas*^*V12*^ or *Kras*^*V12*^ enhanced baseline autophagy in baby mouse kidney epithelial cells,^454^ and this finding was echoed by a later report that autophagic flux was elevated in KRAS-driven PDAC cell lines, which benefits the proliferation of *KRAS*-mutant tumor cells. Further pharmacological inhibition of autophagy by chloroquine has profoundly prolonged the survival of mice with *KRAS* mutant PDAC.^455^ The observed autophagy dependency of mutant RAS-driven cancer cells provides a strong rationale to inhibit autophagy in *RAS*-mutant tumors. In 2019, two publications further advanced this field. Interestingly, they both identified that inhibiting KRAS or its downstream signaling pathways increased the level of autophagy in PDACs, which serves as a protective mechanism for PDACs to support a high proliferative state.^456,457^ Combination of ERK inhibitor or MEK inhibitor with chloroquine synergistically inhibited tumor progression in murine tumor models. Noteworthy, a pancreatic tumor patient had a partial response with 50% reduction in tumor burden following treatment with trametinib plus hydroxychloroquine, while there was no obvious evidence of concurrent toxicity.^457^ As such, cooperatively targeting autophagy and MEK/ERK is a promising therapeutic strategy for treating *KRAS* mutant pancreatic tumors, as well as advanced gastrointestinal malignancies (NCT05221320), biliary cancer (NCT04566133), NSCLC (NCT04735068), and gastrointestinal cancer (NCT04214418) (Table 3).

#### Targeting mutant RAS-driven metabolic alteration in tumor cells

It has been known that tumor cells require a higher metabolic demand, including increased glucose to support their uncontrolled proliferation. Activating KRAS mutations were found to enhance glucose metabolism in different aspects, such as promoting the expression of glucose transporter GLUT1 to increase the uptake of glucose,^458^ inducing activity of key enzymes in glycolysis to increase the glycolytic flux, and supplying bioenergetic intermediates for other anabolic pathways.^459–463^ In addition, KRAS-driven PDAC was found to process glutamine in a non-canonical way mediated by glutaminase (GLS), aspartate transaminase 1/2, malate dehydrogenase 1 (MDH1), and malic enzyme 1 (ME1), which is essential for tumor cells to maintain redox state and proliferation. Glutamine is first catalyzed to glutamate by GLS in mitochondria, and glutamate-derived aspartate in mitochondria is then transported into the cytoplasm. Cytosolic aspartate is then transformed to oxaloacetate by GOT1, and oxaloacetate is further converted to malate and pyruvate via MDH1 and ME1, respectively. Ablation of any participating enzyme would induce dramatic suppression of tumor growth both in vitro and in vivo.^464^ Furthermore, lung adenocarcinoma cells bearing *KRAS*^*G12V*^ mutation displayed enhanced glutathione metabolism mainly triggered by the up-regulation of SLC7A11, which is responsible for importing cystine from the extracellular environment and promoting the synthesis of glutathione to sustain its specific metabolic fitness.^465^ Based on this finding, SLC7A11 inhibitor HG106 effectively inhibits the SLC7A11/glutathione axis and thus results in metabolic lethality in *KRAS*-mutant LUAD.^465^ In addition to altered glucose and glutamine metabolism, *KRAS* mutant NSCLC cancer cells aberrantly activate lipid biosynthesis and β-oxidation through Acyl-CoA synthetase long-chain family member 3 (ACSL3) mediated conversion of fatty acids into fatty Acyl-CoA esters. ACSL3 suppression reduces cellular ATP levels and impairs survival in *KRAS* mutant NSCLC cells.^466^ In addition to ACSL3, the expression of fatty acid synthase (FASN), the rate-limiting enzyme of fatty acid synthesis, was also identified to be upregulated in *KRAS* mutant lung cancer cells.^467^ In line with previous findings, FASN-mediated fatty acid synthesis and subsequently oxidized phospholipid remodeling through the Lands cycle were found to play essential roles in protecting mutant lung cancer cells from ferroptosis.^468^ FASN pharmacological inhibition using TVB-3664 exhibits anti-tumor activity in *KRAS*^*G12D*^-driven lung cancer mouse model in vivo.^468^ Given that these distinct metabolic pathways are dispensable for normal cells, these interesting findings help define new approaches for exploiting the metabolic vulnerability of KRAS-driven cancer. Besides metabolic rewiring driven by oncogenic small GTPases such as KRAS, a recent study reported a reciprocal regulation of small GTPase RAC1 activity by a key metabolic enzyme LDHA through a catalytically independent mechanism.^469,470^ LDHA was found to bind to RAC1 and block RAC GAP access and maintain RAC1 in its active GTP bound state, leading to constitutive RAC1 activation and malignant progression and metastasis of breast cancer. This study links the noncanonical function of metabolic enzymes to small GTPases regulations, which opens a new avenue for studying the crosstalk between rewired metabolism and small GTPases signaling and foster identification of new therapeutic strategies targeting the interface between metabolic enzymes and small GTPases.

### Resistance mechanisms and overcoming strategies

#### Resistance to KRAS^G12C^ inhibitor and combination Strategy

The distribution of KRAS mutations differs among various cancer types and notably, *KRAS*^*G12C*^ prevails in about 41% of lung adenocarcinomas,^471,472^ as well as in 3% of colorectal and 1% of pancreatic cancer.^473–475^ In the past two years, the FDA granted approval for two KRAS^G12C^ inhibitors, Sotorasib and Adagrasib, to NSCLC patients with *KRAS*^*G12C*^ mutation who have received at least one prior systemic therapy, demonstrating the dawn of the clinical application of KRAS^G12C^ inhibitor.^278,476^ Despite the impressive efficacy of KRAS^G12C^ inhibitors with around 30-45% response rate and nearly 7-month median progression-free survival in patients with *KRAS*^*G12C*^ mutant lung cancer, therapeutic responses in the clinic are usually short-lived, attributed to emerging resistance to monotherapy. The latest result of the first phase III trial of KRAS^G12C^ inhibitor Sotorasib in NSCLC patients with KRAS^*G12C*^ mutation (CodeBreak 200 study, NCT04303780) showed that 12-month treatment of Sotorasib pronouncedly improves the progression-free survival (PFS) with 24.8% and overall response rate (ORR) with 28.1% comparing to docetaxel, whose PFS is 10.1% and ORR is 13.2%. However, improvement of overall survival was not observed in this trial.^477,478^ Multiple studies revealed intrinsic, adaptive, and acquired resistance to KRAS^G12C^ inhibitors, and it is urgent to decipher resistance mechanisms and develop rational combinatorial treatment approaches to prevent the emergence of drug resistance to KRAS^G12C^ inhibitors.

**Fig. 8:**
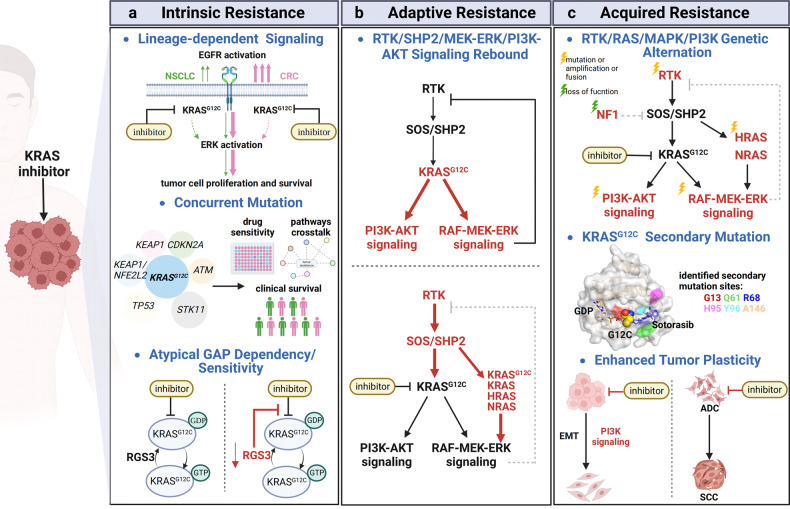
Intrinsic, adaptive, and acquired resistance to KRAS^G12C^ inhibitors. Resistance of KRAS^G12C^ inhibitors can be classified as (**a**) intrinsic, (**b**) adaptive, and (**c**) acquired resistance. **a** Lineage specific signaling between different tumor types, concurrent gene mutations with KRAS oncogenes, atypical GAP dependency/sensitivity have been reported to drive intrinsic resistance to KRAS^G12C^ inhibitors. **b** ERK signaling and PI3K/AKT signaling rebound driven by strong reactivation of different nodes such as RTK, SOS/SHP2, wild type RAS, or KRAS^G12C^ is the main cause of adaptive resistance to KRAS^G12C^ inhibitors. **c** Acquired genetic alterations in RTK, NRAS, HRAS, RAF, MEK, loss of function mutation of GAPs such as NF1, secondary mutations of *KRAS*^*G12C*^ including G13, Q61, R68, H95, Y96, and A146, as well as enhanced tumor plasticity including EMT and histologic transformation are associated with the acquired resistance to KRAS^G12C^ inhibitors. This figure was created with BioRender.com

The underlying mechanisms of intrinsic resistance to KRAS^G12C^ inhibitors [Fig. 8a] remain elusive. Genetic heterogeneity in different types of tumors and co-occurring mutations have been recently identified to confer intrinsic resistance toward KRAS^G12C^ inhibitors.^479,480^ Deep next-generation sequencing was launched in total of 330 NSCLC patients with KRAS mutations, and a panel of the most frequently concurrent mutations including *TP53* (41%), *STK11* (28%), *KEAP1/NFE2L2* (27%), and *KEAP1* (24%) were identified.^481^ Later, exploration of the effect of *TP53*, *STK11* and *KEAP1* co-mutation on the response of NSCLC patients with *KRAS*^*G12C*^ mutation to KRAS^G12C^ inhibitor was initiated (NCT03600883).^482^ The lowest response rate was observed in a group of wild-type *STK11* and mutated *KEAP1*, whereas the highest response was found among patients with mutated *STK11* and wild type *KEAP1*. However, the potential effects of STK11 and KEAP1 that modulate the clinical responses to KRAS^G12C^ inhibitor remain unclear since the number of patients included in co-occurring genomic alteration analysis was limited. Further analysis is ongoing in a phase Ib/II clinical trial of KRAS^G12C^ inhibitor, in *KRAS*^*G12C*^ mutant NSCLC harboring mutant *STK11* and wild-type *KEAP1* (NCT05276726). Upon KRAS activation, cyclin D/CDK4/6 complex get activated, and subsequently phosphorylates retinoblastoma (RB) and promotes tumor cells’ entry into S phase. KRAS^G12C^ inhibitor administration mainly arrests tumor cells in G0/G1 phase. Combined treatment with the CDK4/6 inhibitor further blocks escaping cells in the G1/S checkpoint, leading to enhanced cell cycle arrest and tumor regression.^479^ Clinical trials for combination of G12C inhibitors and CDK4/6 inhibitors are in progress (NCT04185883; NCT04165031). RGS3, a previously known GAP of G-protein signal transduction, was recently identified to mediate hydrolysis of GTP in KRAS mutants, which inactivates KRAS and provides the rationale for the antitumor efficacy of GDP bound KRAS^G12C^ inhibitor.^51^ Further validation in PDX models and patient samples demonstrated that RGS3 is negatively correlated with KRAS mutant signaling output, and patients who have lower RGS3 expression showed resistance to the therapy of G12C inhibitor. This exciting finding unveils a new mechanism for modulating the GTPases activity of KRAS mutants and suggests that RGS3 may serve as a possible biomarker to guide informed therapeutic strategy.

Adaptive resistance limits the clinical benefit of almost all targeted therapies, and this has also limited the efficacy of G12C inhibitors^51,289,452,475,483–485^ [Fig. 8b]. A much lower response rate was observed in colorectal cancer than in NSCLC following treatment with KRAS^G12C^ inhibitor. Higher ERK signaling rebound driven by strong reactivation of EGFR limits the efficacy of KRAS^G12C^ inhibitors in *KRAS*^*G12C*^ mutant colorectal cancer. This finding motivates the development of combination therapy for colorectal cancer by co-targeting both KRAS^G12C^ and EGFR.^486,487^ Because EGFR/SHP2 signaling is a well-known upstream stimulus for activation of KRAS^G12C^, co-targeting EGFR/SHP2 signaling and KRAS^G12C^ is an attractive strategy. A recent study reported a divergent response in *KRAS*^*G12C*^ lung cancer cells following KRAS^G12C^ inhibitor treatment through single-cell RNA sequencing.^452^ KRAS^G12C^ inhibition rendered some cells in a quiescent state whereas others rapidly escape inhibition. Drug-induced quiescent cells produce new KRAS^G12C^, which can be activated by EGFR. Active AURK signaling further promotes KRAS-effector activation, which leads to adaptive cell cycle progression in the presence of the drug. Another study found activation of WT HRAS and NRAS by feedback relief through multiple RTKs as an alternative mechanism for the adaptive resistance to KRAS^G12C^ inhibitors in colorectal cancer cells,^488^ which is consistent with the previous findings that the two remaining WT RAS isoforms (NRAS and HRAS) also play crucial roles in promoting RTK/MAPK signaling activation and cell growth in KRAS mutant-driven tumor cells.^489^ This study also found combining KRAS^G12C^ inhibitors with an SHP2 inhibitor exhibited better anti-tumor efficacy in colorectal tumor PDX models than the KRAS^G12C^ inhibitor and EGFR inhibitor combination, largely because SHP2 inhibition can block RAS reactivation mediated by multiple RTKs upon KRAS^G12C^ inhibition.^488^ Therefore, to overcome adaptive resistance, combinatory therapies of G12C inhibitors with EGFR inhibitor (NCT04185883) and SHP2 inhibitor (NCT04185883) are currently under clinical evaluation.

Aside from its role in conferring adaptive resistance to KRAS^G12C^ inhibitors, RAS-MAPK signaling reactivation was also reported to contribute to acquired resistance to KRAS^G12C^ inhibitors in human lung cancer patients. In an old male patient with KRAS^G12C^-mutant lung adenocarcinoma, the initial response was rapidly observed just a few days after taking Sotorasib, and some measurable tumors were noted to decrease in size. However, at week 13 post-treatment, some lesions began to grow, indicating the emergence of acquired resistance. At week 17, Sotorasib was stopped due to rapid tumor progression.^490^ Along with the rapid development of drug resistance, heterogeneous resistant mechanisms involving reactivation of MAPK and PI3K-AKT-mTOR signaling, epithelial-to-mesenchymal transition (EMT), immune evasion, metabolic rewiring, loss of *KRAS*^*G12C*^ allele and copy number were observed in multiple lesions derived from different sites in this patient.^490^ Meanwhile, in a 67-year-old patient with metastatic *KRAS*^*G12C*^-driven NSCLC treated with Adagrasib,^483^ *KRAS*, *NRAS*, *BRAF,* and *MAP2K1* mutations emerged in the cell-free DNA of this patient after disease progression and all led to the reactivation of RAS-MAPK signaling [Fig. 8c]. Of note, *KRAS*^*G12C/Y96D*^ secondary mutation was found to disrupt Adagrasib binding, which was sufficient to retain KRAS activation in the presence of Adagrasib.^483^ Other secondary mutations in SII-P of KRAS discovered in a clinical study cohort (NCT03785249), including R68S, H95D, H95Q, H95R, and Y96C, were reported to confer resistance to Adagrasib in vitro^483^ [Fig. 8c]. Interestingly, H95D, H95Q, and H95R mutations still showed marked response to Sotorasib treatment, implying that different drug-binding modes between Adagrasib and Sotorasib can potentially induce drug-specific resistance mutations.^289^ Hotspot mutations in Ras family or BRAF, such as *KRAS*^*G12V*^, *NRAS*^*Q61K*^, *MRAS*^*Q71R*^, and *BRAF*^*G596R*^ were illustrated to have a strong propensity to bypass KRAS^G12C^ inhibition and drive resistance via reactivating ERK signaling, which was found in 43 paired specimens obtained before and after the treatment of Sotorasib.^484^ Consistently, co-targeting ERK with G12C inhibitors led to an enhanced anti-tumor activity both in resistant cell models and PDX models.^484^ Additionally, distinct oncogenic fusions involving *BRAF*, *ALK*, *RET* and *FGFR3* also emerged in progressive tumors from patients treated with Adagrasib.^289^ Thus, mutations in the MAPK pathway genes, as well as oncogenic mutations and fusions in RTK, constitute the acquired resistance to G12C inhibitors, and combinatorial inhibition of KRAS^G12C^ mutant and these cooperative targets such as RTKs (NCT04185883; NCT03785249; NCT04165031), MEK(NCT04185883), and RAF dimers (NCT05074810) has been proven to be an effective strategy to overcome resistance.

EMT has been found to trigger adaptive as well as intrinsic resistance to KRAS^G12C^ inhibitors in NSCLC cell lines^485^ [Fig. 8c]. EMT induces sustained activation of PI3K/AKT and reactivation of ERK in NSCLC tumor cells during the treatment of Sotorasib. Hence, the addition of PI3K inhibitor GDC-0941 into the cell lines can restore the sensitivity of Sotorasib. Triple combination of Sotorasib, GDC-0941 with SHP2 inhibitor SHP099 exhibits a more pronounced anti-tumor effect than Sotorasib dual combination with either GDC-0941 or SHP099.^485^ Moreover, a histologic transformation from adenocarcinoma to squamous cell carcinoma was discovered in two out of ten patients (nine NSCLC patients and one colorectal tumor patient) who developed acquired resistance after the treatment with KRAS^G12C^ inhibitor [Fig. 8c]. Intriguingly, no resistant mechanisms previously reported at the genomic level were found within these two patients (NCT03785249).^289^ Since KRAS mutations are rarely observed in squamous cell carcinoma.^491^ It is possible to speculate that this histological transformation may serve as an alternative way to escape the blockade of KRAS^G12C^ inhibitor.

#### Emerging targeting strategies

Since preclinical studies reported that KRAS^G12C^ inhibitor demonstrated greater efficacy in tumor growth inhibition in immuno-competent mice when compared with immune-deficient mice,^492^ it is vital to develop novel strategies to combine RAS inhibitors with immunotherapies to achieve greater clinical benefit for patients with RAS-mutants. It is well established that KRAS mutants contribute to the immunosuppressive tumor microenvironment through multiple mechanisms,^493–496^ including prompting M1 to M2 polarization of macrophages, inducing regulatory T cell differentiation by secreting TGF-β and IL-10, enhancing infiltration of myeloid-derived suppressor cells, and disrupting CD8^+^ cytotoxic T cells activation through modulation of immune checkpoint signaling. Therefore, combining targeted KRAS mutant inhibition with immune checkpoint blockade (ICB) is a straightforward strategy that has shown synergistic antitumor effects in the CT26 tumor xenograft model^492^ [Fig. 7b]. Meanwhile, adoptive cell therapies targeting KRAS mutants,^497,498^ tumor vaccines^499^ and antibodies targeting KRAS mutant peptide-MHC I complex^500^ are now in full swing [Fig. 7b]. Lately, a proof-of-concept study identified that neoantigen derived from ARS1620-mediated covalent modification of KRAS^G12C^ could be presented by MHC I in cancer cells. Antibodies recognizing these neoepitopes were developed and displayed immunotherapeutic efficacy in *KRAS*^*G12C*^ tumor cell lines.^501^ Remarkably, ARS1620-targeted antibody triggered cytotoxic T-cell response in tumor cell lines with intrinsic or acquired resistance to ARS1620 when co-cultured with peripheral blood mononuclear cells,^501^ demonstrating the potential to overcome drug resistance. Concurrently, a HapImmune^TM^ platform was established to discover antibodies targeting the Sotorasib-engaged KRAS^G12C^ peptide/MHC complexes.^502^ R023 scDb, which was developed by combining HapImmune^TM^ and T cell-engaging bispecific antibody platform, can selectively kill Sotorasib-pre-treated KRAS^G12C^ mutant lung cancer cells that show intrinsic resistance to Sotorasib.^502^ Lastly, Ras-driven cell transformation has also been reported to be dependent on the activity of Rho, Arf, and Ran proteins, which provides a strong rationale to target Ras, Rho, Arf, and Ran to synergistically suppress the proliferation of RAS-driven tumor cells and reverse RAS mediated malignant phenotype.^503–505^

Collectively, the approval of KRAS^G12C^ inhibitor ignited the enthusiasm of developing allele-specific inhibitors targeting RAS, and now the major direction of the pharmaceutical industry is developing inhibitors directly targeting different alleles of RAS. Nevertheless, exploring approaches that indirectly target RAS including its upstream regulators, downstream effectors, synthetic lethal partners, metabolic reprogramming, immune microenvironment, and signaling crosstalk is equally important since they are the most promising candidate targets to be applied in combinatorial therapies to enhance efficacy of direct RAS targeting inhibitors and combat drug resistance.

#### Concluding remarks

Since the establishment of association with human diseases, the journey of targeting small GTPases family has been laborious but also fulfilling. As a predominant oncogenic protein, KRAS has been established as a paradigm in the process of drug discovery towards small GTPases, with numerous targeting strategies developed during the recent decade. On the contrary, the efforts to target Rho, Arf, Rab, and Ran families are lagging and most of the identified compounds remain insufficiently characterized and lack follow-up optimizations. In 2021, Sotorasib received accelerated approval from the FDA to treat NSCLC harboring *KRAS*^*G12C*^ mutation during its phase II clinical trial. Now, this drug has been approved in 44 markets, including the United States, the European Union, the United Kingdom, and Japan. This has been reckoned as a milestone in proving the druggability of KRAS after its discovery 40 years ago. In 2022, AMGEN announced results from the global Phase 3 CodeBreaK 200 trial. 25% of patients showed PFS compared with 10% for chemotherapy reagent docetaxel, while OS between Sotorasib (10.6 months) and docetaxel (11.3 months) remaining unchanged. Treatment-related adverse events are 33% for Sotorasib relative to 40% for docetaxel. As such, the clinical performance and adverse effects are still under evaluation for further approval.^38,506^ Also in 2022, J&J announced they are terminating further clinical trials of another KRAS^G12C^ covalent inhibitor JNJ-74699157 (ARS-3248) due to “dose-limiting skeletal muscle toxicities and the lack of efficacy at the 100 mg dose”.^280^ This shows the adverse effects observed during clinical trials and its underlying mechanisms need to be further addressed. Also during 2022, the new ligands that selectively bind to KRAS G12D, G12S, and G12R mutants have been developed, renewing the frontier to target alleles-specific hotspot mutations of KRAS.^506^ The G12D inhibitor exhibits encouraging antitumor efficacy in PDAC tumor models^38^ and G12S/R inhibitors provide proof of concept approaches to inspire the future development of other KRAS mutants.

Despite the successful clinical application of KRAS^G12C^ inhibitors in NSCLC patients with *KRAS*^*G12C*^ mutations, resistance develops in almost all patients. Uncovering the sophisticated mechanisms of intrinsic, adaptive, and acquired drug resistance will be the key to developing novel combinatory strategies to target cancers driven by RAS mutations, which will facilitate the process of targeting other RAS isoforms and other small GTPases. KRAS^G12C^ inhibitors induced a quiescent state in a subpopulation of tumor cells and these quiescent cancer cells synthesized new KRAS^G12C^ mutant protein, escaping drug inhibition through EGFR and AURKA-mediated RAS signaling activation.^452^ This study explained why KRAS^G12C^ inhibitor treatment only had partial responses in most lung cancer patients. Further mechanistic studies into the divergent responses following KRAS^G12C^ inhibitor treatment and transcriptional modulations controlling new KRAS synthesis in drug-induced quiescent cells will uncover new therapeutic targets and modalities to overcome adaptive resistance to current KRAS^G12C^ inhibitors.^452^ KRAS inhibitors often trigger a cytostatic response in cancer cells and targeting a quiescent cell state will strengthen tumor targeting and likely lead to tumor cell death to achieve durable and complete responses in cancer patients. Acquired resistances of KRAS^G12C^ inhibitors are engendered by KRAS secondary mutations or fusions/mutations occurring at upstream RTKs or downstream RAF/MEK. 10 distinct alterations in KRAS, NRAS, BRAF, and MEK were identified in a KRAS^G12C^ NSCLC patient with acquired resistance to Adagrasib.^483^ In another clinical study, more than one potential resistance mechanism was identified in 41% (7/17) of patients with KRAS^G12C^ NSCLC or CRC.^289^ As such, the heterogeneity of resistance mechanisms poses challenges to both basic research and clinical treatments. Since it is difficult to target multiple heterogenous resistant mechanisms at the time of tumor relapse, we need to find a way to prevent the expansion of tumor heterogeneity along with KRAS^G12C^ inhibitor treatment. Mechanistic studies into the evolution of heterogenous resistant alterations under drug pressure through combining single-cell DNA sequencing and barcode tracing techniques will improve our understanding of the expansion of tumor heterogeneity and identify potential targets to delay the emergence of the acquired resistance.

Further exploration into uncovering new regulatory mechanisms underlying biological functions of small GTPases and developing mechanism-based targeting strategies are still needed for targeting different small GTPases and their hotspot mutations. Liquid-liquid phase separation (LLPS) offers a new angle to decipher the complex regulatory mechanisms in cells.^507,508^ The cytoplasmic tail of EGFR and GRB2 (Growth factor receptor-bound protein 2) form a binary condensate upon the phosphorylation of EGFR, and then LLPS recruits SOS at the membrane surface for Ras activation.^509,510^ RTK fusion proteins can condensate into de novo cytoplasmic membraneless granules, which further recruit and concentrate Grb2/SOS for initiating Ras-mediated MAPK signaling.^511^ A parallel study reveals that RTK forms condensate at the membrane surface and this process is critical in recruiting SHP2 and PLCγ1 and modulating their enzymatic activities.^512^ Other small GTPases such as Rac and Rab are also implicated in mediating LLPS.^513,514^ As introduced in the first session, PTMs play unique roles in modulating the structures and functions of small GTPases. Because they are highly diversified and heterogeneous, they remain largely undiscovered. Therefore, the continuous discovery of new PTMs and functional/structural characterizations will bring new insights into targeting regulation mechanisms. For instance, the finding of a dynamic process of NRAS phosphorylation mediated by STK19 led to the discovery of STK19 inhibitor to target NRAS mutant melanoma.^424,426^ Increasing efforts are also directed at defining new mediators of the oncogenic properties of Ras. A recent study identified EFR3A as a new interaction partner for oncogenic KRAS through mining interactomes of oncogenic Ras proteins. EFR3A binds specifically to active KRAS and further recruits phosphatidylinositol kinase PI4KA.^515^ This leads to the accumulation of PI4P and phosphatidylserine (PtdSer) at the plasma membrane, which promotes KRAS plasma membrane localization, nanoclustering, and activation of downstream signaling. Moreover, a selective PI4KA inhibitor showed synergistic anti-proliferative activity with KRAS^G12C^ inhibitors in KRAS^G12C^ mutant human cancer cell lines.^515^ Another study uncovered the essential role of PtdSer lipid transport proteins in maintaining PM PtdSer levels and KRAS PM localization, and further showed targeting PI4KIIIα to reduce PM PI4P and PtdSer levels inhibited KRAS-driven tumor growth in pancreatic cancer models.^516^ Recently, a new protein RASON encoded by long noncoding RNA (LINC00673) is reported to lock KRAS^G12D^ and KRAS^G12V^ in a GTP-bound state and thus lead to continuous KRAS activation.^517^ In the PDAC xenograft model, deletion of RASON sensitizes KRAS mutant PDAC to EGFR inhibitor and inhibits tumor growth. As such, identifying novel proteins that maintain/accelerate small GTPases activity might define more vulnerable targets to block small GTPases’ oncogenic signaling alternatively. The spatiotemporal dynamics of the subcellular distribution, transient dimerization, and switching between active/inactive conformations are not only important for the biological functions of GTPases but also useful for inferring mechanisms of drug actions. Although in-situ real-time visualization and quantification of these dynamic properties are still challenging, versatile engineered fluorescent biosensors and single molecular methods have been developed for measuring these properties of heterotrimeric G-protein^518–520^ and small GTPases^521–525^ in living cells.

Strategic innovations by adopting or developing new methods for targeting small GTPases are in high demand. Combinatory therapies of KRAS inhibitors with inhibitors of the upstream regulators or downstream effectors have been proven with enhanced anti-tumor activities. Targeting crosstalk between different small GTPases is another interesting avenue because accumulating evidence indicates that the transformation of RAS-driven tumor cells requires endogenous activities of Rho proteins or overexpression of Rho, Arf, and Ran.^526^ KRAS^G12C^ inhibitors have been reported to drive a pro-inflammatory tumor microenvironment and enhance the anti-tumor activity when combined with immune checkpoint blockade (ICB).^492,527^ However, a recent study indicates that G12C inhibitor selectively synergizes with ICB in inflamed tumors but not in non-inflamed tumors, which addresses a strong immunogenicity dependency of synergizing KRAS^G12C^ inhibitor with ICB and guides the selection of patients who might respond to this combination.^528^ Further mechanistic studies into the failure of KRAS^G12C^ inhibitors to synergize with ICB in non-inflamed tumors will be needed to provide novel strategies to improve the therapeutic effect of combined KRAS^G12C^ inhibition and ICB.

Instead of combining KRAS^G12C^ inhibitors with ICB, another attractive direction is developing bispecific T cell engagers targeting KRAS mutant peptide-MHC I complex on the surface of KRAS^G12C^ mutant cancer cells.^501,502^ Currently, these bispecific T cell engagers based on a KRAS^G12C^ inhibitor conjugated neoepitopes have been reported to cause cytotoxic T cells response in a co-culture system with T cells in vitro. Cancer cells need to be pretreated with a covalent KRAS^G12C^ inhibitor for a period to allow full engagement of the drug with KRAS^G12C^ mutant protein and its subsequent presentation by MHC. The dosing schedule of the covalent KRAS^G12C^ inhibitor and the antibody needs to be optimized to achieve maximum targeting efficiency. Further, in vivo bioavailability, safety profiles, and immunotherapeutic effects of these antibodies targeting KRAS^G12C^ inhibitors-based neoepitopes are waiting to be evaluated to move forward to its clinical application. Taken together, KRAS^G12C^ inhibitor-targeted immunotherapy or cotreatment of KRAS^G12C^ inhibitor with immunotherapy represents new directions in tackling drug resistance to KRAS^G12C^ inhibitors through remodeling the immunosuppressive tumor microenvironment driven by KRAS mutants.

Multi-omics have been undergoing fast evolution, which acts as a powerhouse for mapping gene regulatory networks at the cellular level and uncovering concerted targets at transcriptional and post-transcriptional levels. However, large-scale data outputs pose challenges in data mining and interpretation for the protein of interest, and more specific functional validations and characterizations are necessary. Protein structure is pivotal in drug design and complex structures of small GTPases with their regulatory proteins or downstream effectors are limited. This issue might be improved with the recent developments of cryoEM for structural determinations and deep learning methods for structural predictions such as AlphaFold Multimer^529^ and RoseTTA Fold.^530^ Understanding the structural plasticity of switch regions in small GTPases is a unique resource for developing inhibitors with desirable selectivity to the disease mutants. The exploitation of structural dynamics and lowly populated conformations for drug discovery has been promoted for years^531,532^ and has been attested by some of the recent studies on KRAS, such as CLAMPs,^327^ NMR-derived ensembles of the switch pockets^49^ and allosteric regulations.^533^ Now, it is time for bringing this concept from the bench to the bedside with more energetic efforts. New computational platforms using artificial intelligence for ultra-large-scale virtual screening are reshaping the workflow of drug discovery. They can unprecedentedly maximize the chemical space and elevate the time efficiency in drug screening.^534–537^ Recently, the Random nonstandard Peptide Integrated Discovery (RaPID) system has been successfully employed to screen macrocyclic peptide inhibitors for G-protein (Gαs).^538^ Two macrocyclic peptides are cell-permeable and nucleotide-state-selective; they can bind to the GDP- and GTP-bound Gαs by distinguishing the conformation of Switch II/helix-3 pocket. RaPID is established by combining mRNA display technique with in vitro translation system^539,540^ and the library diversity of macrocyclic peptides can reach a scale of 1000 molecules. Both Gα and small GTPases belong to the family of GTP-binding proteins and share a structural similarity, which foresees the potential applicability of RaPID to screen for macrocyclic peptide inhibitors of small GTPases. PROTACs are evolving on a fast track and will play a more important role in eliminating the disease mutants with the future development of more specific chemical compounds and protein ligands. Targeting Ras membrane association through PI4KA inhibitors represents a new indirect targeting strategy and holds promise for KRAS mutant cancer patients.^515,516^ Enhanced dependence on lipid biosynthesis and β-oxidation of KRAS mutant cancer cells also leads to new approaches to target the metabolic vulnerability of KRAS-driven cancers.^466–468^

In summary, many methodological innovations and monumental progresses have been made in targeting KRAS. However, the mission of drug discovery for small GTPases is far from being completed. For a protein family with over 150 members, there are only two drugs targeting KRAS G12C approved in the clinic. For KRAS^G12C^ inhibitors, further efforts are needed to improve its PFS rates, and combat drug resistance and adverse effects. Less progress has been made in targeting NRAS^Q61^ mutation for treating melanoma and other hotspot mutations (G12V, G13, Q61H/L) have not yet been targeted. G12V mutation occurs in both KRAS and CDC42 and it is intractable by current targeting strategies because the sidechain of valine is less chemically amenable than others. Most of the KRAS inhibitors currently in clinical trials are targeting GDP-bound state by preventing KRAS activation. Future efforts are summoned to establish effective strategies to target the GTP-bound active conformation for directly turning off the oncogenic mutants. Progress of targeting Rho, Arf and Rab falls far behind Ras. Cross-activity is a practical concern raised during the development of isoform-specific ligands for Rho, Rab and Arf family proteins due to high sequence similarity and shared regulatory proteins within each subfamily. Discerning the subtle structural difference that potentially leads to uncovering different pocket properties and finding chemical reactive residues for covalent binding are keys to circumventig this obstacle, and a plethora of lessons can be learned from targeting KRAS. Therefore, drug discovery for Rho, Arf, Rab, and Ran should be illuminated rather than overshadowed by Ras, and the uncharted space for academic research and industry is enormous.
